# A new preclinical sheep model of medial meniscus anterior root repair: Part 1—Quantitative morphology and relationships to adjacent structures

**DOI:** 10.1002/ksa.12656

**Published:** 2025-03-25

**Authors:** Wei Liu, Marta Carretero‐Hernández, Yin Zhang, Magali Cucchiarini, Matthias Brockmeyer, Henning Madry

**Affiliations:** ^1^ Center of Experimental Orthopaedics Saarland University Homburg Germany; ^2^ Department of Orthopaedics and Orthopaedic Surgery Saarland University Medical Center Homburg Germany

**Keywords:** lateral meniscus roots, medial meniscus roots, menisci, sheep, tibial plateau

## Abstract

**Purpose:**

To analyse the quantitative morphology of the menisci, their roots and relations with a focus on the medial meniscus anterior root (MAR) as a basis for a preclinical model.

**Methods:**

Data was obtained from 24 tibial plateaus of skeletally mature, female Merino ewes. The MAR attachment (MARA) was scanned with micro‐computed tomography and stained with hematoxylin and eosin. Data of relevant anatomical structures was subjected to principal component analysis (PCA) and Spearman correlations.

**Results:**

The osteo‐ligamentous junction of the MARA represents a classical enthesis with a type‐I insertion into the cortical bone. The medial tibial plateau was of a significantly smaller area than lateral. Its sagittal length was significantly longer than lateral. The widths of the MAR and lateral meniscus anterior root (LAR) were approximately half of both anterior horn widths. The MAR was significantly wider than the LAR. The medial meniscus body, posterior horn and medial posterior root were significantly thinner than lateral. PCA and cluster analysis revealed a striking, significant distinction between the structures of the medial and lateral tibial plateau. The sagittal length of the articular cartilage of both tibial plateaus correlated with the primary axis length of both menisci. The maximum width of the articular cartilage of both plateaus correlated with the area of both menisci. Significant correlations also existed between the length of the MAR and the total width of the tibia plateau and between the size of the MARA and the coronal distance to the medial tibial eminence (MTE), to the tibial tuberosity and the sagittal distance to the MTE.

**Conclusion:**

The ovine MAR may be appropriate for repair approaches because of its morphological similarities to the human situation. The substantial differences between the medial and lateral tibial plateau have to be respected.

**Level of Evidence:**

Not applicable.

**Abbreviations**
ACL: anterior cruciate ligament
AHW: meniscus anterior horn width
AIML: anterior inter‐meniscal ligament
ALB: anterolateral bundle
AMB: anteromedial bundle
ANOVA: analysis of variance
Ar: aspect ratio
ARH: Meniscus anterior root length
ARW: Meniscus anterior root width
BA: bare area
BMD: bone mineral density
BW: Meniscus body width
Circ: circularity
EDL: extensor digitorum longus muscle
EDLG: extensor digitorum longus groove
HE: hematoxylin and eosin staining
LAR: lateral meniscus anterior root
LARA: lateral meniscus anterior root attachment
LBA: lateral bare area
LM: lateral meniscus
LPR: lateral meniscus posterior root
LTE: lateral tibial eminence
LTP: lateral tibial plateau
LTPrim: lateral tibial plateau rim
MAR: medial meniscus anterior root
MARA: medial meniscus anterior root attachment
MBA: medial bare area
Micro‐CT: micro‐computed tomography
MM: medial meniscus
MPR: medial meniscus posterior root
MTE: medial tibial eminence
MTP: medial tibial plateau
MTPrim: medial tibial plateau rim
MTT: medial aspect of the tibial tuberosity
OA: osteoarthritis
PC: principal component
PCA: principal component analysis
PHW: meniscus posterior horn width
PRW: meniscus posterior root width
ROM: range of motion
TE: tibial eminence
TP: tibial plateau
TPrim: tibial plateau rim
TT: tibial tuberosity

## INTRODUCTION

The meniscal roots are of eminent importance for a normal knee function. Root tears result in loads equivalent to a total meniscectomy, are challenging to treat [3, 8, 21] and represent a critical risk factor for osteoarthritis (OA) [1, 4, 17, 21, 43].

Large animal models advance our understanding of knee anatomy, pathology, repair and regeneration [5], including the menisci [37, 44] and their roots [9, 24]. The sheep is the closest to humans in terms of cross‐species similarities of the tibial plateau microstructure [31]. Understanding the topographic anatomy of the ovine medial meniscus anterior root (MAR) is essential when developing models of root repair or meniscus replacement. One of our goals is to propose a sheep model of MAR repair. While the surgical anatomy of the human [18, 23, 24, 25, 26, 30, 42] and rabbit medial meniscus roots and their relation to adjacent structures is well known [12, 16, 17] and lapine models of medial meniscus anterior and posterior root release and repair have been established [14, 17], the quantitative relationship of the ovine MAR to neighbouring structures has not been comprehensively studied to date.

The objective of the present study was to analyse the surgical anatomy of the ovine tibial plateau, medial and lateral meniscus, their roots and their relations as a basis for developing a preclinical model of MAR repair.

## MATERIALS AND METHODS

### Animals

Anatomical data were obtained from 24 knee (stifle) joints (both left and right knees) of 12 skeletally mature, female Merino ewes (2–4 years of age) collected as part of unpublished studies. Sacrifice was in agreement with local and national legislation on the protection of animals and NIH Guidelines for the Care and Use of Laboratory Animals (registration and approval number: 2.4.2.2–22‐2023, Saarland).

### Analysis of the macroscopic anatomy

The analysis of the topographic anatomy was performed by two observers (W. L. and Y. Z.) using photomicrographs taken with a solid‐state CCD camera (Olympus). All photographing was conducted from the same relative position to ensure consistency, and equipped with a scale bar provided from the same ruler allowing for the further evaluation. Measurements were taken directly from the images using ImageJ 1.53 K (National Institutes of Health) to quantify the morphological characteristics. A detailed analysis of the macroscopic anatomy of the sheep's tibial plateau with focus on the MAR attachment (MARA) was measured and its topographic relation to surrounding relevant anatomic structures was assessed, such as: the tendon of the extensor digitorum longus muscle (EDL), the extensor digitorum longus groove (EDLG), the lateral meniscus anterior root (LAR), the LAR attachment (LARA), the anteromedial (AMB) and anterolateral bundle (ALB) of the anterior cruciate ligament (ACL), the medial and lateral meniscus body (MM or LM), the medial or lateral tibia plateau (MTP or LTP), the medial or lateral tibial eminence (MTE or LTE), the border of the tibial plateau (TPrim) and the tibial tuberosity (TT). The following measurements of the topographic anatomy were performed (all data given in mm): distance between MARA and EDLG, between MARA and TT, between MARA and anterior medial bundle (AMB) footprint, between anterior border of medial tibial plateau (MTP) and AMB footprint, between MARA and LAR, sagittal length of MTP/lateral tibial plateau (LTP)/tibial plateau (TP), coronal length of medial tibial eminence (MTE)‐MTP rim (MTPrim)/LTE‐lateral tibial plateau rim (LTPrim)/tibial plateau (TP), the sagittal/coronal distance of MARA‐MTE, and the sagittal/coronal distance of MARA‐LTE. The following areas were determined: MARA, EDLG, MTP, LTP, MBA (medial bare area) and LBA (lateral bare area) (all data given in mm²). The resulting parameters were calculated: circularity (circularity = 4π(area/perimeter^2^; a circularity value of 1 indicates a perfect circle, while if it approaches 0, it indicates an increasingly elongated polygon), the primary and secondary axis (an ellipse was fitted to the selection and the primary and secondary axis of the best fitting ellipse was determined to be the length of the major and minor axis), the aspect ratio of the MARA (ratio of the length between the primary and the secondary axis). In addition, the anterior root width (ARW), anterior horn width (AHW), meniscus body width (BW), posterior horn width (PHW), and posterior root width (PRW) of the MM and LM were measured (all data given in mm).

### Micro‐computed analysis of the MARA

After removing the tibial plateau from the leg with an oscillating saw, the MAR was separated from the anterior horn by sharp dissection and a bone block was established with an oscillating saw with a margin of 5 mm from the macroscopic visual fibres of the root. Bone blocks (*n* = 10) were fixed in 4% formaldehyde (24 h) and scanned in a micro‐computed tomographic (micro‐CT) machine (Bruker Skyscan) (conditions: 90 kV voltage, 278 µA current, 18 µm pixel size, Cu+Al filter, 0.41 rotation steps, three frame rate average, 180° rotation). Images were reconstructed using SkyScan software NRecon (version 1.7.0.4) (parameters: histogram limits of −0.000023, 0.020154, ring artefact 15, beam hardening 30). Gamma images were coloured using the default colour scale from the software SkyScan DataViewer (version 1.5.2.4). The gamma images obtained were then analysed with SkyScan CTAn software (version 1.16.4.1) to determine the thickness of the cortical bone, the bone mineral density (BMD), and to create a 3‐dimensional model. To measure cortical bone thickness *n* = 6 pictures from each region were analysed (*n* = 20 measurements per picture, perpendicular to the bone surface).

### Histological descriptive analysis of the medial meniscus anterior root: attachment, ligament mid‐substance and transition into the horn

Six bone blocks containing the MARA were decalcified and embedded in ParaPlast X‐tra (Leica Biosystems). The respective menisci were dissected to obtain their MAR mid‐substance while keeping its transition into the anterior horn. Additionally, six lateral and medial menisci were sectioned (coronal) at the anterior horn, pars intermedia and posterior horn. Histological sections (3 µm thickness) were stained with hematoxylin and eosin (HE) in a Harris hematoxylin bath (Carl Roth) (10 min), rinsed with water and then bathed in eosin solution (Carl Roth) (40 s), then rinsed again with water. Images were taken with an Olympus SC50 Camera and CellSens Software (v1.18 Standard; Olympus).

### Statistical analysis, correlations

Data are given as mean and standard deviation or median and interquartile range for each separate experiment. The Shapiro–Wilk normality test and the Brown–Forsythe test were employed to check for normal distribution and variance equality. The Kruskal–Wallis test were conducted for nonparametric analysis and Dunn's test for multiple comparisons. The ordinary one‐way analysis of variance (ANOVA) and Brown‐Forsythe ANOVA were performed for parametric analysis and Šídák's test and Dunnett's T3 test for multiple comparisons. Principal component (PC) analysis (PCA) was performed based on a standardized method of PCs selection which was based on a parallel analysis. Cluster analysis was executed with KMeans Clustering [32] with one minus spearman rank correlation. Correlation analysis was calculated with Spearman correlation. *p*‐Values and adjusted *p*‐values were reported with *p* < 0.05 considered statistically significant. Box plot diagrams always showed the interquartile range (upper and lower borders of the boxes), the minimum and maximum (whiskers), the mean value (+), the median (middle line), and the individual data points (dots). All statistical analysis were performed with Prism v.10.2.3 (GraphPad).

## RESULTS

### Macroscopic topographic anatomy of the ovine MAR and its attachment (MARA)

**Figure 1:**
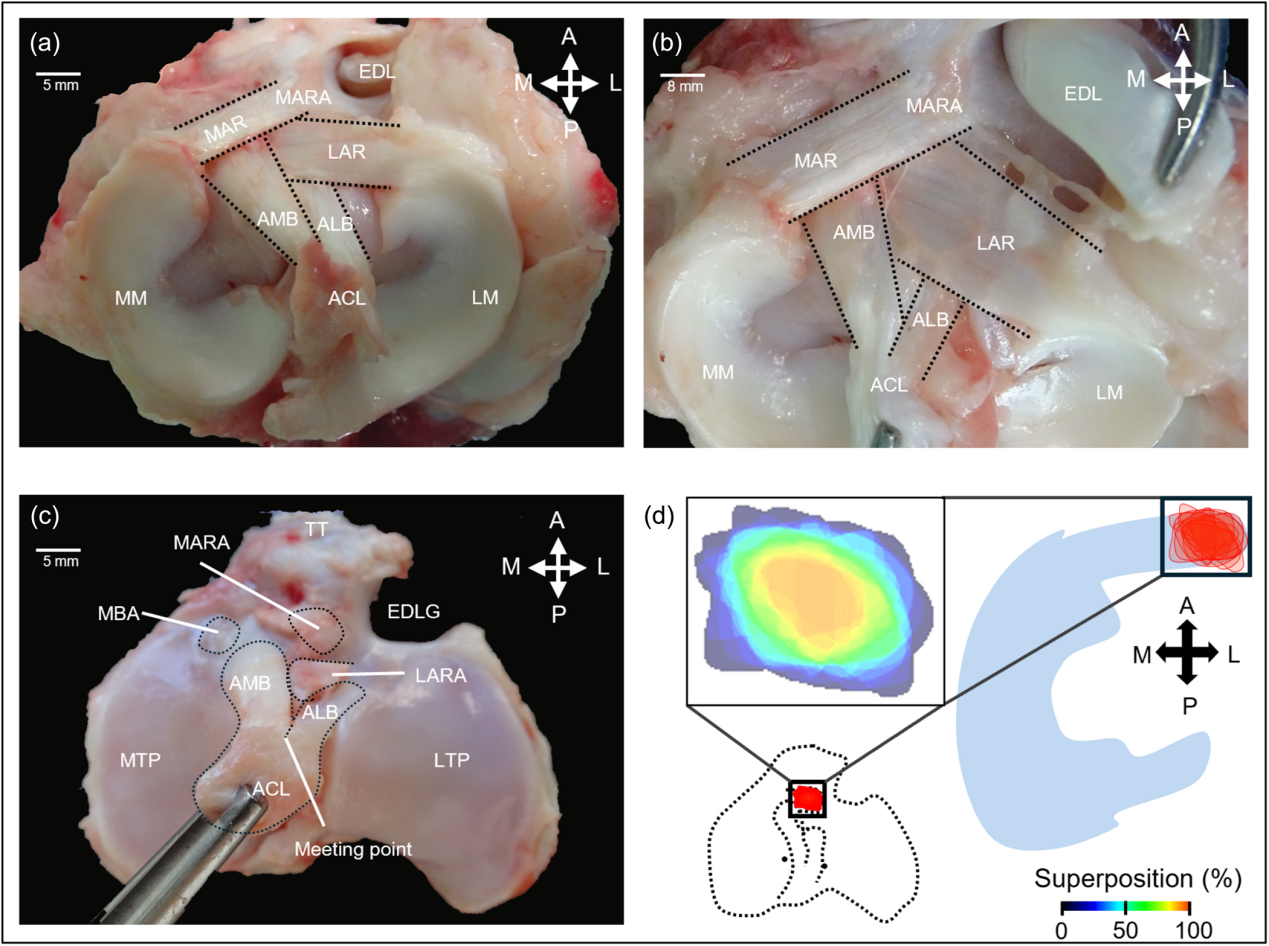
Macroscopic view of the ovine medial meniscus anterior root (MAR). (a) Photographic view in proximo‐distal direction of the region around the MAR. (b) Closer proximo‐distal view of the MARA and LARA with surrounding structures. (c) Proximo‐distal view of tibial plateau upon removing the menisci and their roots. The ovine anterior cruciate ligament (ACL) consists of an antero‐medial (AMB) and an antero‐lateral bundle (ALB). The attachment of the AMB lies in close proximity to the MARA. (d) The area of MARA is of elliptical shape (red colour; larger images, inset shows the individual shapes and overlapping boundaries of the MARA, *n* = 24). EDL, tendon of the extensor digitorum longus muscle; EDLG, extensor digitorum longus groove; LAR, lateral anterior root; LARA, lateral anterior root attachment; LM, lateral meniscus; LTP, lateral tibial plateau; MARA, medial meniscus anterior root attachment; MM, medial meniscus; MTP, medial tibial plateau; TT, tibial tuberosity.

The ovine MAR consists of a single distinct ligamentous bundle (root ligament) that inserts at the site of the MARA (Figure 1a). The MAR is well distinguishable from the anterior horn (Figure 1b). It traverses the tibial plateau in an antero‐medial direction. A variable anatomy of the MAR as in humans or common fibres with other ligaments were not observed (*n* = 24). The MAR is surrounded by multiple adjacent structures that overlap in part. Anterior to the MARA lies the TT. Medial posterior to the MARA is the tibial attachment of the AMB of the ACL, while the AML of the ACL lies posterior to the LAR. Both bundles converge into the ACL posterior to the LAR. The MARA is anterior to the LARA, and anterolateral to it lies the EDL groove, containing the tendon of the EDL. Posterolateral lies the attachment of the LAR. Removing the MAR exposes the area of the MARA (Figure 1c). It has an elliptical shape (*n* = 24) (Figure 1d). Immediately medial to the MARA, a fibrocartilaginous zone below the MAR that has a spatial relationship to the medial aspect of the insertion of the AMB of the ACL emerges (Figure 1c). Medially adjacent to it, a bare area characterized by the absence of fibrocartilage or articular cartilage continues to lie below the root. The articular cartilage surface then commences medial to it, below the medial meniscus anterior horn.

### Microstructural analysis of the ovine MARA

**Figure 2:**
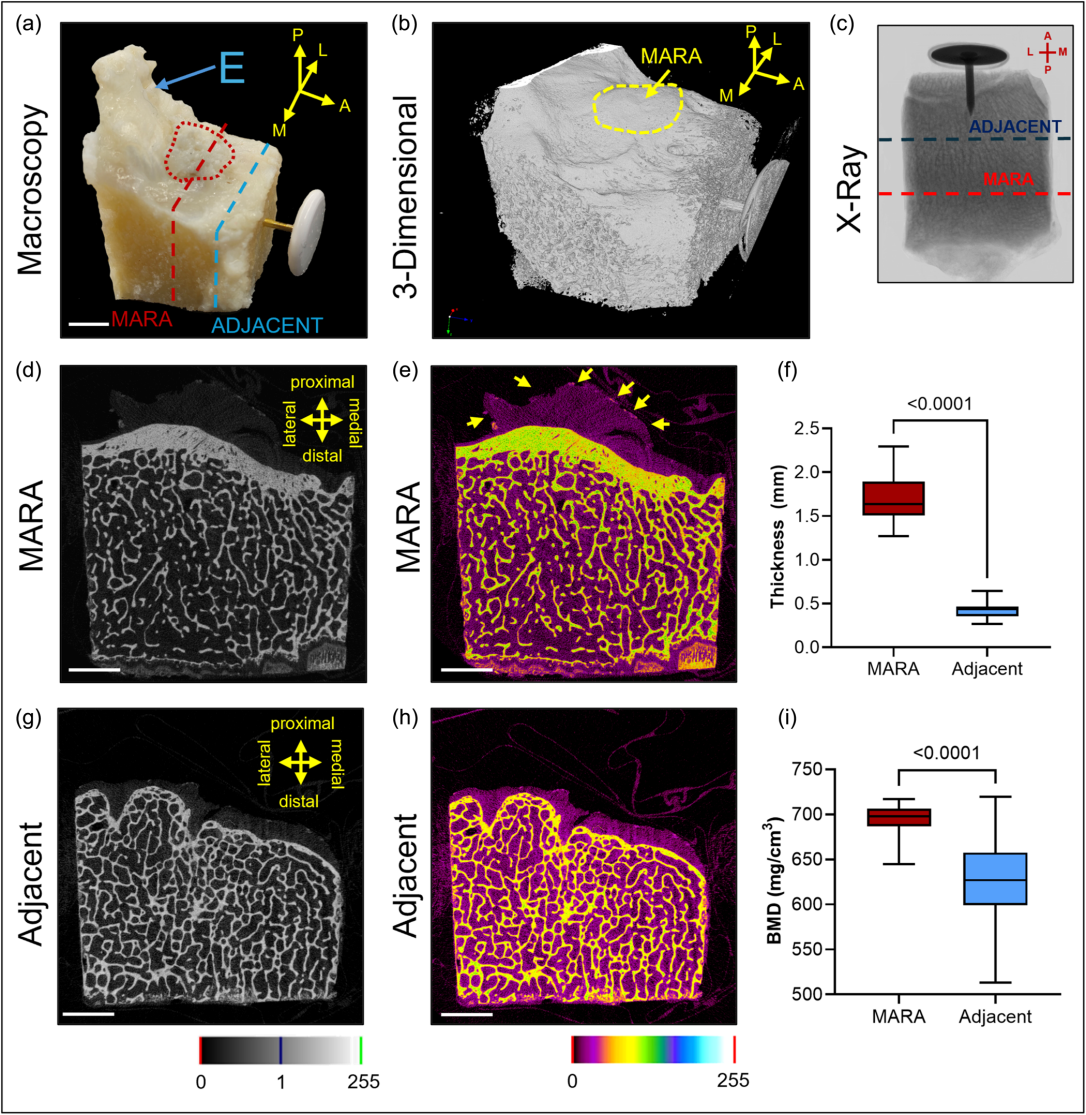
Micro‐computed tomographic (micro‐CT) structure analysis of the ovine medial meniscus root attachment (MARA). (a) MARA and adjacent tibial plateau bone specimen including the intercondylar eminence (E: eminentia). The interrupted red line marks the sectioning of MARA, the interrupted blue line marks the sectioning of the adjacent tibial bone. (b) Micro‐CT 3‐dimensional reconstruction, the area of the MARA is highlighted. (c) Transversal X‐ray image. (d, e) Gamma coronal image of the MARA and its fibres (yellow arrows) and (f, g) of the adjacent bone. (h) Cortical bone thickness (mm) of the bone below the MARA and adjacent bone. (i) Bone mineral density (BMD) of the cortical bone below the MARA and the adjacent bone. The metallic pin in a, b, and c marks anterior. Scale bars: (a) 10 mm; (d, e, g, h): 5 mm.

Micro‐CT analysis of the MARA revealed an undulated enthesis zone on the cortical bone surface (Figure 2d,e). In contrast, the adjacent cortical bone did not show an even surface and a specific structure (Figure 2g,h). The centre of the MARA exhibited a peak in both thickness and topographic height (Figure 2d,e), being higher topographically than the AMB of the ACL enthesis cortical bone. The cortical bone of the MARA was about 4x larger than the adjacent cortical bone (1.69 ± 0.29 and 0.43 ± 0.09 mm, respectively. *p* < 0.0001) (Figure 2f). The BMD of the cortex bone below the enthesis was significantly higher than the anterior adjacent cortex bone (696.10 ± 14.00 and 628.80 ± 47.90 mg/cm^3^, respectively, *p* < 0.0001) (Figure 2i).

### Histological analysis of the ovine MARA, ligament mid‐substance and transition into the horn

**Figure 3:**
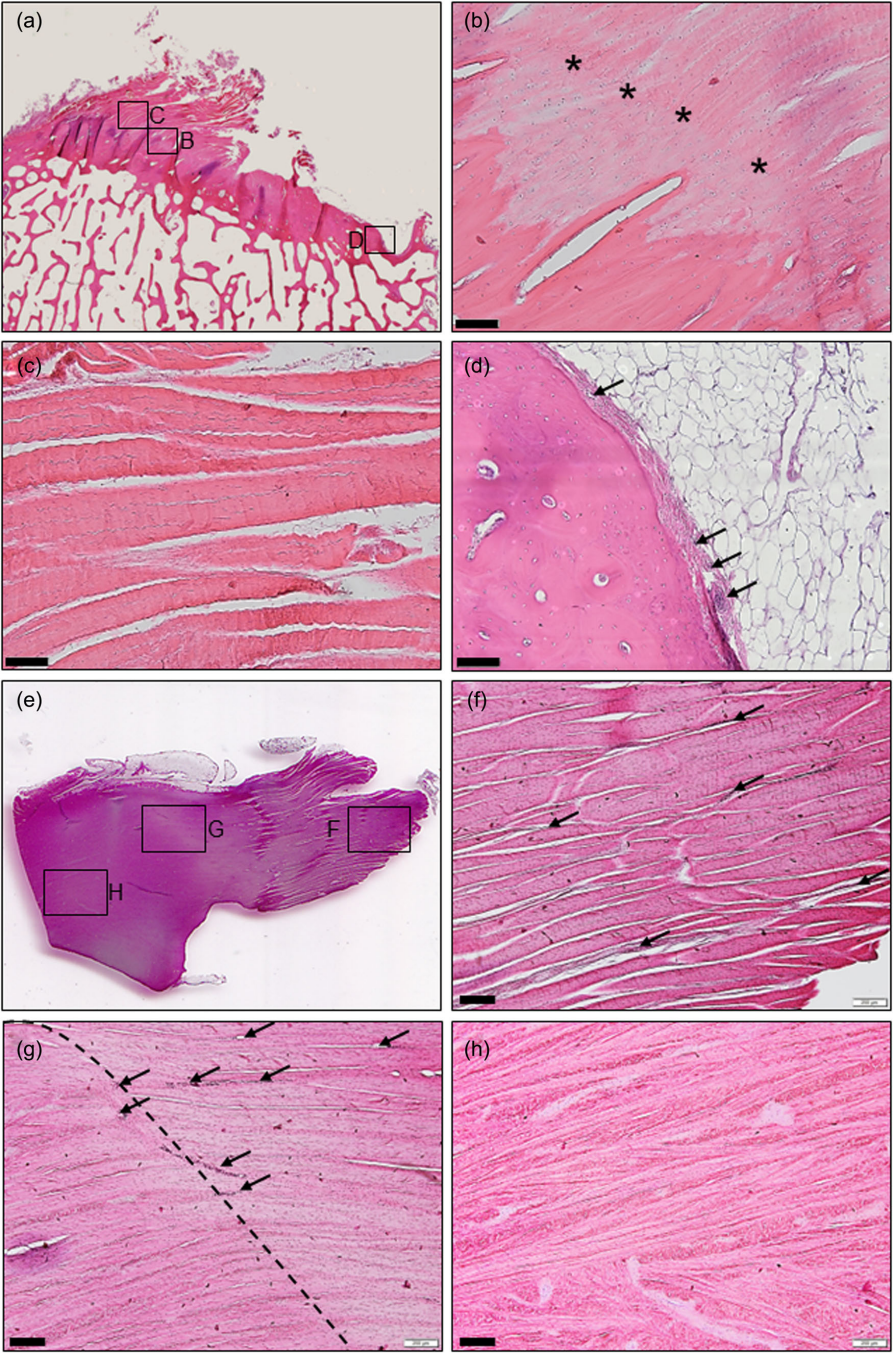
Histological analysis of the medial anterior meniscus root (MAR) attachment, ligament mid‐substance and its transition into the horn. (a) Coronal overview of the MAR bony insertion. (b) Fibrocartilage at the bony insertion of the MAR. (*) Tidemark. (c) Closest ligament fibres to the attachment site. (d) Detail of the medial bare area, blood vessels (arrows) are between the bone and the adipocyte cover layer. (e) Transversal overview of the dissected MAR and the anterior horn. (f) Detail of the ligament mid‐substance, rich in blood vessels (arrows), organized in thick fascicles. (g) Transition (dash line) from the ligament (right) into the horn (left). Blood vessels (arrows) predominant in the ligament side. (h) Fibre organization in the meniscus horn where transversal and axial fibres are intercalating. Scale bars: (b–d) 100 µm; (f–h) 200 µm.

At the MARA, no blood vessels were observed in the fibre insertion into the bone (Figure 3a). Round, chondrocyte‐like cells were found at the MARA and surrounding them the eosin staining appeared weaker, similar to that of fibrocartilaginous tissue. This fibrocartilaginous tissue showed a well‐defined cement line and tidemark (Figure 3b). The fibrocartilaginous fibres transition into pure ligament fibres packed in thick fascicles (Figure 3c). The layer between the bone tissue and adipocytes in the bare area located medially to the insertion showed vascularization (Figure 3d). The ligament mid‐substance was rich in blood vessels (Figure 3f). They disappeared when transitioning into the meniscus horn (Figure 3g), remaining in less abundance at the most peripheral region of the horn, the red‐red zone. The fibre organization changed considerably when reaching the transition zone, where the fibres from the ligament mid‐substance unweaved and mixed in thinner bundles, intercalating circumferential and radial fibres in the horn (Figure 3g,h).

### Histological analysis of the ovine medial and lateral menisci

**Figure 4:**
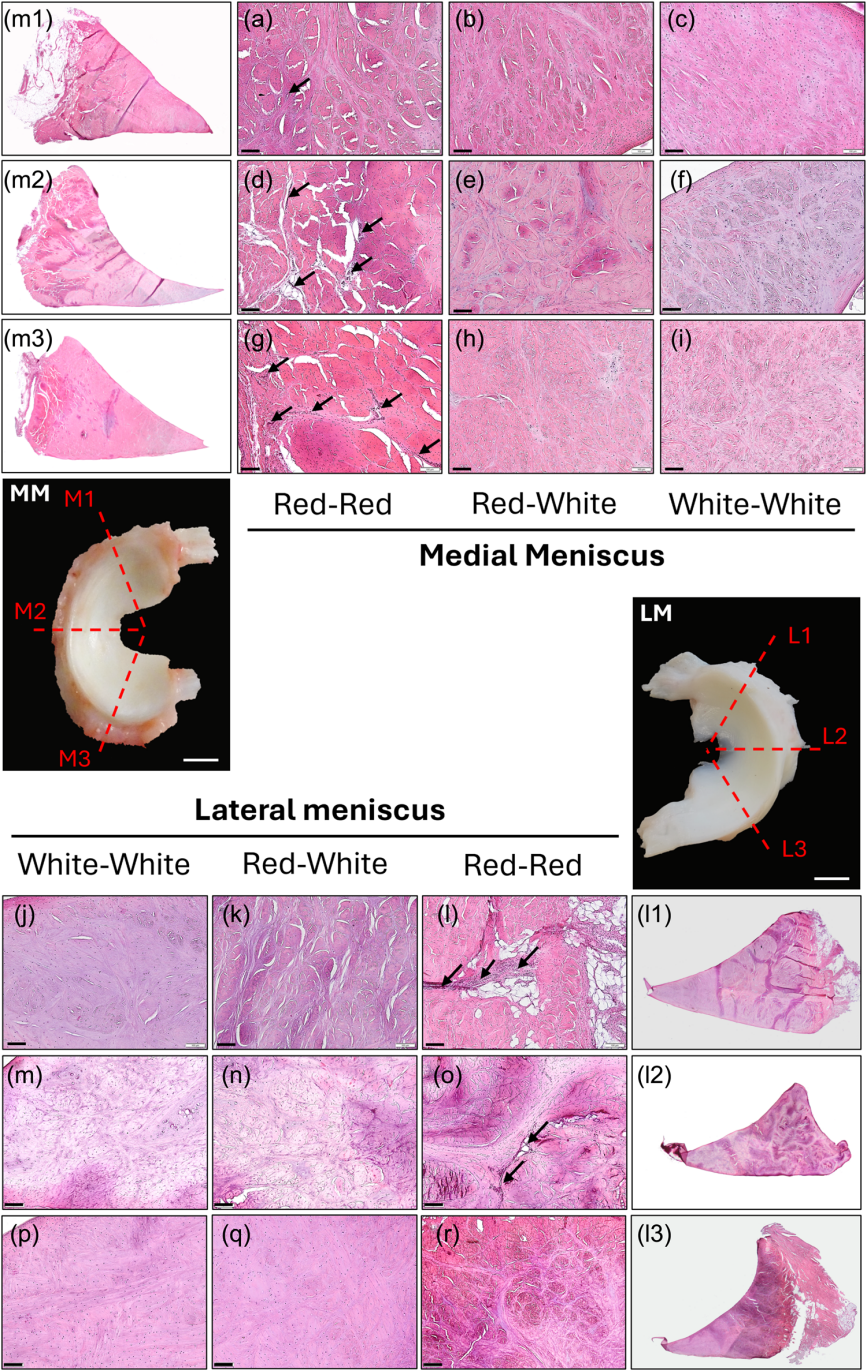
Histological staining with hematoxylin and eosin of the different regions of the lateral and medial meniscus. Blood vessels only visualized in red‐red zones, pointed with arrows. Chondrocyte‐like cells presence in red‐white and white‐white zones. (m1, a–c) Medial meniscus (MM) anterior horn. (m2, d–f) MM pars intermedia. (m3, g–i) MM posterior horn. (l1, j–l) Lateral meniscus (LM) anterior horn. (l2, m–o) LM pars intermedia. (l3, p–r) LM posterior horn. Scale bars: MM, LM: 1 cm; a–r: 100 µm.

The medial meniscus pars intermedia showed an increase in the curvature on its femoral side, being steeper than the anterior and posterior horns (Figure 4m1–m3). The LM region with the highest curvature was the posterior horn, while the anterior and pars intermedia showed a more triangular shape (Figure 4l1–l3). The red‐red zone was characterized by an abundance of blood vessels that penetrated the meniscus body from the adipose tissue in the periphery, and bundles of circumferential fibres with fusiform cells (Figure 4a,d,g,l,o,r). The red‐white zone presented areas of HE staining with round chondrocyte‐like cells. These regions were connected by fibres of a radial orientation, slightly lower eosin‐stained than the circumferential fascicles (Figure 4b,e,h,k,n,q) The eosin staining continued to decrease until it reached the white‐white zone, characterized by a lack of blood vessels, abundance of chondrocyte‐like cells and a fibre structure of intercalating circularly and axial fibres (Figure 4c,f,i,j,m,p).

### Analysis of the relationship of relevant structures around the medial and LTP

**Figure 5:**
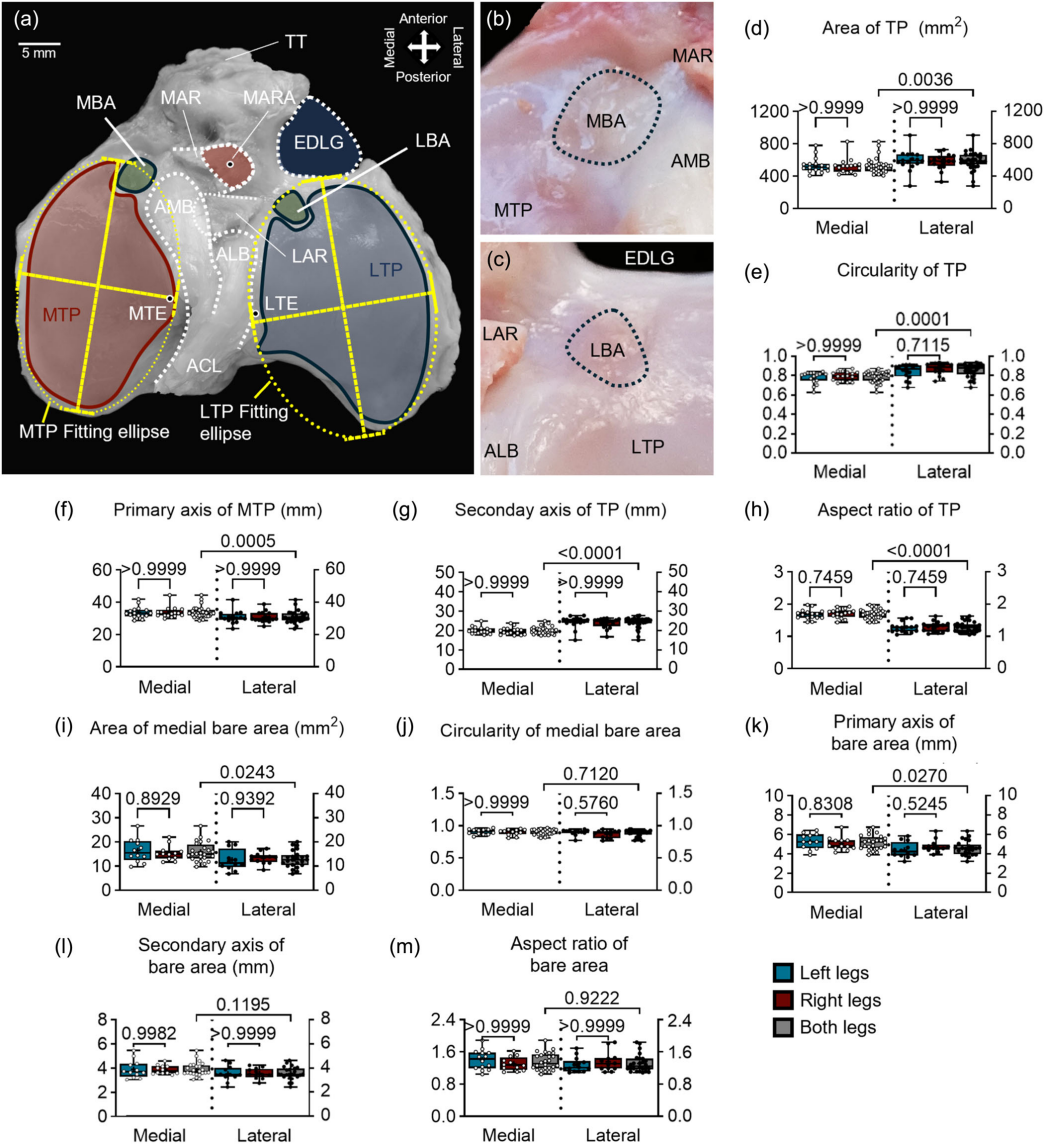
Anatomy analysis of the ovine tibial plateau. (a–c) Tibial plateau and bare area. (d–h) Morphology parameters of tibial plateau. (i–m) Morphology parameters of bare area. ACL, anterior cruciate ligament; ALB, anterolateral bundle; AMB, anteromedial bundle; EDLG, extensor digitorum longus groove; LAR, lateral anterior root; LBA, lateral bare area; LTE, lateral tibial eminence; LTP, lateral tibial plateau; MAR, medial anterior root; MARA, medial anterior root attachment; MBA, medial bare area; MTE, medial tibial eminence; MTP, medial tibial plateau; TT, tibial tuberosity.

Distinct morphological features characterized the tibial plateau (Figure 5a–c). The area of the MTP was significantly (1.12‐fold) smaller than the LTP (525.60 ± 94.87 and 587.40 ± 113.00 mm², respectively; *p* = 0.0036) (Figure 5d). The circularity of the MTP was significantly reduced (0.79 ± 0.05 and 0.85 ± 0.07 mm, respectively; 1.08‐fold difference, *p* = 0.0001) (Figure 5e). The secondary axis of the MTP was significantly shorter in length than the LTP (19.92 ± 1.82 and 24.13 ± 2.86 mm, respectively; 1.21‐fold difference, *p* < 0.0001) (Figure 5g). The primary axis of the MTP was significantly longer (33.73 ± 3.40 and 30.80 ± 3.52 mm, respectively; 1.09‐fold difference, *p* = 0.0005) (Figure 5f) and the aspect ratio was significantly higher than the LTP (1.7 ± 0.15 and 1.29 ± 0.15, respectively; 1.32‐fold difference, *p* < 0.0001) (Figure 5h).

The sizes of the medial and lateral bare areas adjacent to the MARA and LARA were significantly different (Figure 5b,c) 15.90 ± 4.00 and 12.94 ± 3.50 mm², respectively (1.22‐fold difference, *p* = 0.0243) (Figure 5i). The circularities of the medial and lateral bare areas were of 0.90 ± 0.04 and 0.88 ± 0.53, respectively (1.02‐fold difference, *p* = 0.7120) (Figure 5j). The primary axis of the medial bare area was significantly (1.12‐fold) longer than lateral (5.16 ± 0.75 and 4.58 ± 0.75 mm, respectively; *p* = 0.0270) (Figure 5k). The secondary axis was not significantly different (3.89 ± 0.58 and 3.5 ± 0.54 mm, respectively; *p* = 0.1195) (Figure 5l). The aspect ratio of the medial and lateral bare areas was of 1.36 ± 0.21 and 1.31 ± 0.21, respectively (*p* = 0.9222) (Figure 5m). No significant statistical difference existed for the left and right knees for all the parameters.

### Analysis of the MM and LM and their roots

**Figure 6:**
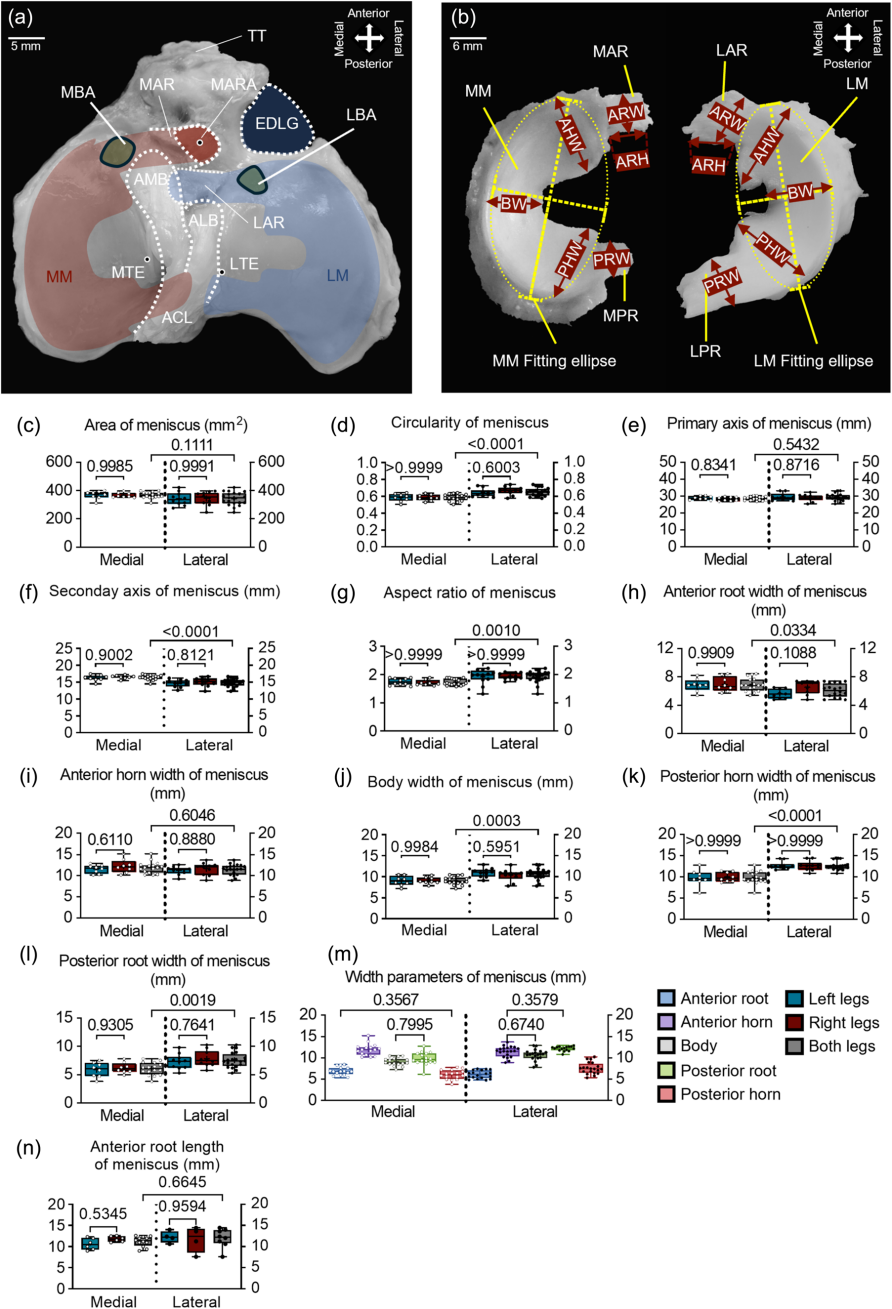
Macroscopic analysis of structures of the ovine menisci. (a) Tibial plateau with marked meniscus location. (b) Macroscopical photo of meniscus. (d–g) Morphology parameters of the menisci. (h–l) The width of different regions of the meniscus. (m) Comparison among the meniscus widths of different regions. (n) The length of the anterior root of the menisci. ACL, anterior cruciate ligament; AHW, anterior horn width; ALB, anterolateral bundle; AMB, anteromedial bundle; ARW, anterior root width; BW, body width; EDLG, extensor digitorum longus groove; LAR, lateral anterior root; LBA, lateral bare area; LPR, lateral posterior root; LTE, lateral tibial eminence; LM, lateral meniscus; MAR, medial anterior root; MARA, medial anterior root attachment; MBA, medial bare area; MM, medial meniscus; MPR, medial posterior root; MTE, medial tibial eminence; PHW, posterior horn width; PRW, posterior root width; TT, tibial tuberosity.

The areas covered by the entire body of the MM and LM (Figure 6a,b) were not significantly different (369.40 ± 22.93 and 343.90 ± 46.32 mm², respectively; *p* = 0.1111) (Figure 6c). The circularities of the MM and LM were significantly different (0.58 ± 0.04 and 0.65 ± 0.08, respectively; 1.11‐fold difference, *p* < 0.0001) (Figure 6d). The primary axis of the medial and LM was not significantly different (28.69 ± 1.18 and 29.40 ± 2.08 mm; *p* = 0.5432) (Figure 6e). The secondary axis of the medial meniscus was significantly longer than of the LM (16.39 ± 0.74 and 14.84 ± 1.20 mm, respectively; 1.10‐fold difference, *p* < 0.0001) (Figure 6f). The aspect ratio of the medial and LM was significantly different (1.74 ± 0.11 and 1.94 ± 0.21; 1.11‐fold difference, *p* = 0.0010) (Figure 6g).

The MAR was significantly wider (1.13‐fold) than the LAR (6.92 ± 0.93 and 6.10 ± 0.93 mm, respectively; *p* = 0.0334) (Figure 6h). The widths of the medial and lateral anterior horn were not significantly different (11.95 ± 1.36 and 11.46 ± 1.23 mm, respectively; *p* = 0.6046 (Figure 6i). The medial meniscus body was significantly thinner (1.17‐fold) than lateral (9.07 ± 0.97 and 10.69 ± 1.29 mm; *p* = 0.0003 (Figure 6j). The medial posterior horn was significantly thinner (1.28‐fold) than lateral (9.85 ± 1.52 and 12.62 ± 1.01 mm; *p* < 0.0001 (Figure 6k). The medial posterior root (MPR) was significantly thinner (1.25‐fold) than lateral (6.06 ± 1.11 and 7.58 ± 1.36 mm; *p* < 0.0019 (Figure 6l). The lengths of the medial and lateral anterior root were not significantly different 11.42 ± 1.26 and 12.01 ± 2.21 mm, respectively; *p* = 0.6645 (Figure 6n). Notably, the width of the MAR was approximately half of the medial meniscus AHW (6.92 ± 0.93 and 11.95 ± 1.36 mm, respectively; 1.73‐fold difference, *p* < 0.0001). The width of the lateral anterior root was also approximately half of the LM AHW (6.01 ± 0.93 and 11.48 ± 1.23 mm; 1.91‐fold difference, respectively, *p* < 0.0001). The width of the MPR was approximately 2/3 of the medial PHW (6.06 ± 1.11 and 9.85 ± 1.51 mm, respectively; 1.62‐fold difference, *p* < 0.0001). The width of the lateral posterior root (LPR) was approximately 2/3 of the lateral PHW (7.58 ± 1.36 and 12.62 ± 1.01 mm, respectively; 1.66‐fold difference, *p* < 0.0001) (Figure 6m). No significant statistical difference was detected between the left and right legs for all the parameters.

### Analysis of the relationship of relevant structures around the ovine MARA

**Figure 7:**
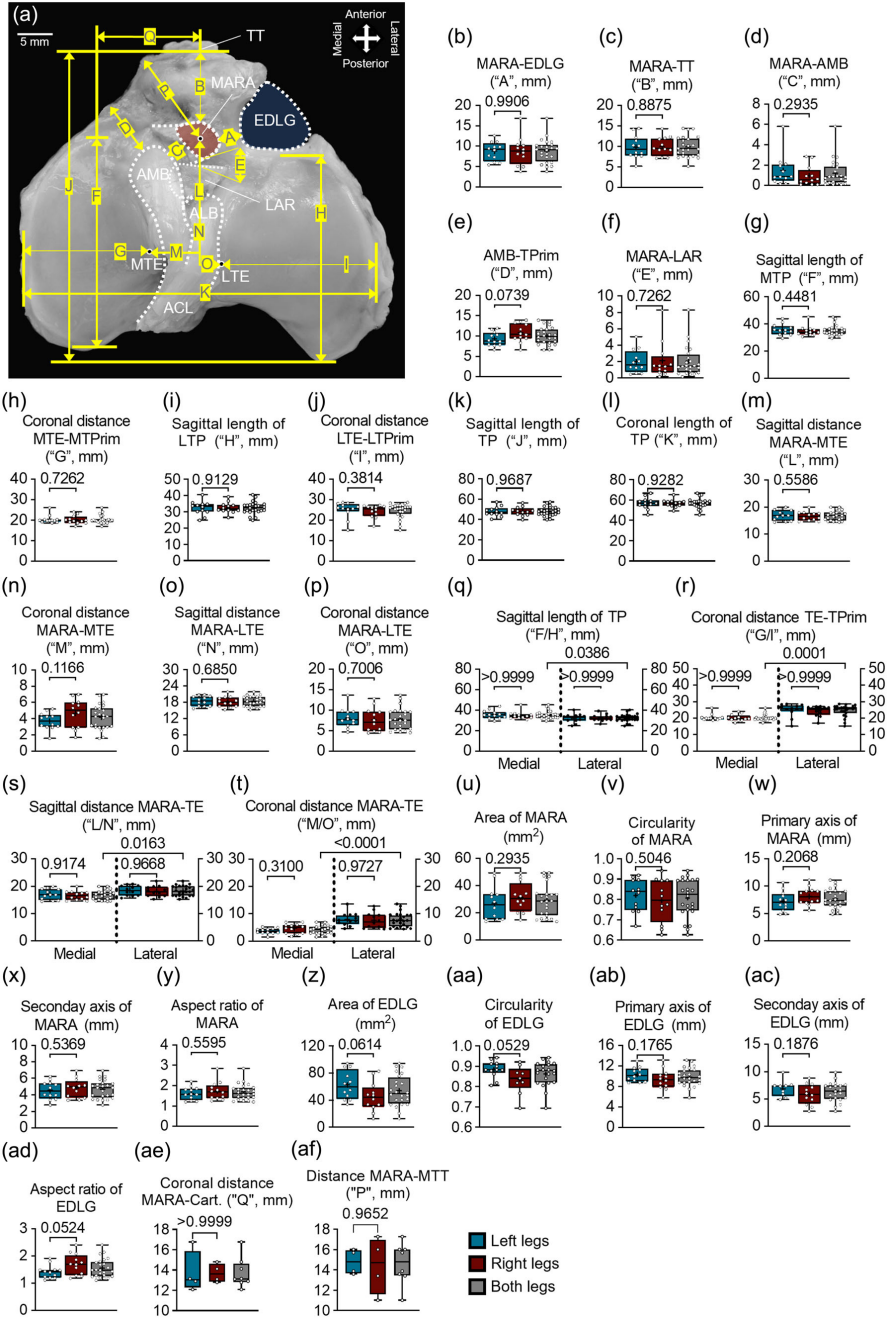
Anatomy analysis of the ovine medial meniscus anterior root attachment (MARA). (a–p) Special parameters around MARA. (q–t) Comparison among a–p parameters. (u–y) Morphology parameters of MARA. (z–ad) Morphology parameters of extensor digitorum longus groove (EDLG). (ae–af) Distances between the medial meniscus anterior root area and the articular cartilage and medial aspect of the tuberosity, respectively. ACL, anterior cruciate ligament; ALB, anterolateral bundle; AMB, anteromedial bundle; LAR, lateral anterior root; LTE, lateral tibial eminence; MAR, medial anterior root; MTE, medial tibial eminence; TT, tibial tuberosity.

The following distances from the MARA (Figure 7a) were determined: 8.80 ± 2.98 mm (Figure 7b) to the EDLG, 9.94 ± 2.50 mm to the TT (Figure 7c), 1.21 ± 1.30 mm to the AMB bundle of the ACL (Figure 7d) and 2.06 ± 1.86 mm to the LAR (Figure 7f). The distance between the AMB bundle footprint and border of the tibial plateau was 10.05 ± 2.12 mm (Figure 7e). Other relevant parameters included the sagittal length of the medial (35.43 ± 3.85 mm) (Figure 7g), lateral (32.41 ± 3.72 mm) (Figure 7i) and entire tibial plateau (48.01 ± 4.49 mm) (Figure 7k), the coronal distance of the MTE to the MTPrim (20.40 ± 1.96 mm) (Figure 7h), the distance between the LTE and LTPrim (24.64 ± 3.48 mm) (Figure 7j) and the coronal width of the entire tibial plateau (56.89 ± 4.79 mm) (Figure 7l). Interestingly, the MTP had a significantly longer sagittal length than the LTP (1.09‐fold difference, *p* = 0.0386) (Figure 7q), opposite for the coronal distance of the tibial eminence border of the tibial plateau (1.20‐fold difference, *p* = 0.0001) (Figure 7r). The sagittal and coronal distances between the MARA and the MTE were 16.86 ± 1.78 and 4.19 ± 1.50 mm (Figure 7m,n). The sagittal and coronal distances between the MARA and the LTE were 18.36 ± 1.85 and 7.84 ± 2.60 mm (Figure 7o,p). Notably, the sagittal length between MARA and the MTE was significantly shorter than between MARA and the LTE (1.08‐fold difference, *p* = 0.0163) (Figure 7s). Significant findings existed for the coronal distance between MARA and the tibial eminence (1.87‐fold difference, *p* < 0.0001) (Figure 7t).

Special morphological features of the MARA included circularity, the primary and secondary axis of the best fitting ellipse, and aspect ratio of axis (Figure 7u–y). Identical parameters were obtained for the EDLG (Figure 7z–ad). Additionally, the distances between the MARA and the anterior edge of the articular cartilage in the MTP (Figure 7ae) and the medial aspect of the TT were measured (Figure 7af). In all the cases, no significant differences exist between all parameters of the left and right knees.

### Multivariate statistics of the relationship of relevant structures around the ovine medial meniscus anterior root attachment, tibial plateau, and meniscus

**Figure 8:**
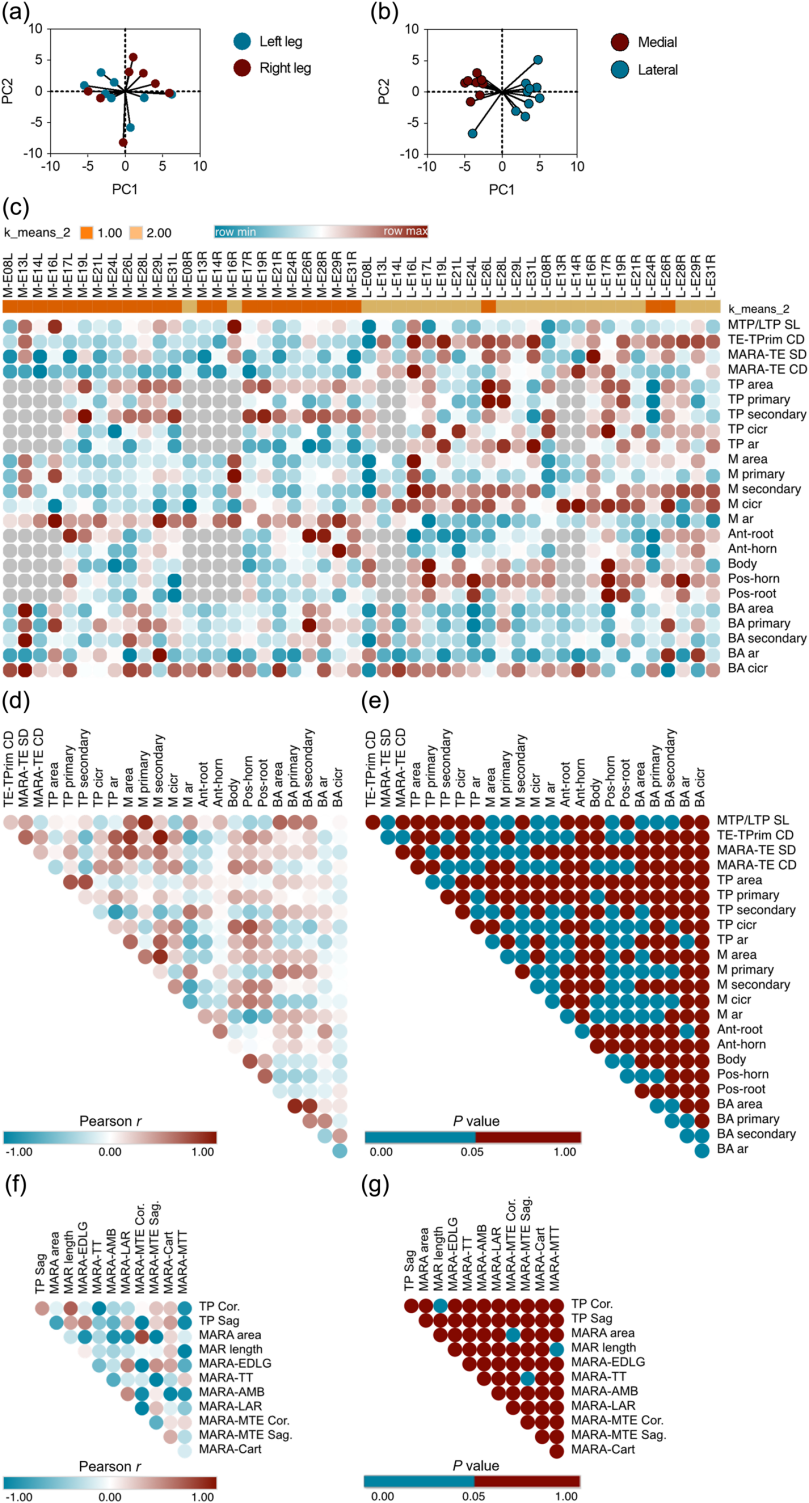
Multivariable analysis of symmetry‐related parameters. (a) PCA between left leg and right leg. (b) PCA between medial and lateral. (c) Cluster analysis for parameters between medial and lateral. (d) Pearson *r* value of correlation analysis for symmetry‐related parameters. (e) *p* value of correlation analysis for symmetry‐related parameters. (f) Pearson *r* value of correlation analysis for relevant anatomical structures in relation to the MAR. (g) *p*‐value of correlation analysis for relevant anatomical structures in relation to the MAR. Ar, aspect ratio; BA, bare area; Cart, articular cartilage; CD, coronal distance; Cicr, circularity; LTP, lateral TP; M, meniscus; MAR, medial anterior root; MARA, MAR attachment; MTP, medial TP; MTT, medial aspect of the TT; PCA, principal component analysis; SL, sagittal length; TE, tibial eminence; TP, tibial plateau; TPrim, tibial plateau rim; TT, tibial tuberosity.

PCA performed on all measured parameters revealed no significant differences between left and right knees of the same animal (Figure 8a). However, PCA indicated a significant distinction between the medial and lateral sides of the tibial plateau based on all the structures measured (Figure 8b), a finding that was further validated by cluster analysis (Figure 8c). The data revealed that the structures of the medial and LTP were strikingly different. There were no morphological side‐to side differences between the structures of the tibial plateau. However, significant strong correlations (all *r* > 0.7) were identified between 7 of the 529 (1.3%) evaluated symmetry‐related parameters (positive: 6; negative: 1). Significant moderate correlations (all *r* > 0.5) were identified between 24 of the 529 (4.5%) evaluated parameters (positive: 18; negative: 6). Of note, the sagittal length of the articular cartilage of both tibial plateaus correlated with the primary axis length of both menisci (*r* = 0.91, *p* < 0.0001), the maximum width of the articular cartilage of both medial and lateral tibia plateaus correlated with the area of both menisci (*r* = 0.85, *p* < 0.0001) and with the secondary axis of the menisci (*r* = 0.94, *p* < 0.0001), and the area of both menisci correlated with their secondary axis (*r* = 0.87, *p* < 0.0001) (Figure 8d,e).

For the MAR, significant correlations were identified between the length of the MAR and both the total width of the tibia plateau (TPCor.) (*r* = 0.75, *p* = 0.032) and the distance to the medial aspect of the tibial tuberosity (MTT) (*r* = −0.73, *p* = 0.037). Further significant correlations were between the size of the MARA and the coronal distance between the MARA and the medial eminence (*r* = 0.83, *p* = 0.01), and between the distance from the MARA to the TT and the sagittal distance between the MARA and the medial eminence (*r* = −0.76, *p* = 0.027) (Figure 8f,g).

## DISCUSSION

The most important morphologic finding is that the ovine MAR may be appropriate for repair approaches because of its morphological similarities to the human situation. The osteo‐ligamentous junction of the MARA represents a classical fibrocartilaginous enthesis with a type‐I insertion into the cortical bone. Both ovine menisci were of similar structure and vascularization as in humans. Remarkably, the widths of the MAR and the LAR were approximately half of both meniscus AHWs. The MAR was significantly wider than the LAR, and the medial meniscus body, posterior horn and MPR were significantly thinner than lateral. Correlation analyses establish the TT and the MTE as key anatomical landmarks to consider in translational surgery when aiming to avoid damage to surrounding structures (AMB of ACL, LAR) of the MAR. The substantial differences between the structures of the medial and LTP have to be respected.

The ovine MARA was always flat, a characteristic defined by Berlet and Fowler as type‐I meniscal attachment [7]. The ovine MARA was anterior to the LARA, while anterolateral in rabbits [12]. The lapine medial meniscus transversed laterally over the top of the lateral anterior root, which was not seen here. The ovine MARA is also anterolateral to the AMB of the ACL, while the lapine MARA is located more anteriorly and slightly more laterally in relation to the tibial ACL footprint [12]. The osteo‐ligamentous insertion of the ovine MARA represents a classical enthesis. The finding that its cortical bone is about 4 times larger than normal cortical bone with a higher BMD (similar to the ovine subchondral bone plate), is explained by the mechanostat theory [19], implying that bone mass and architecture reflect mechanical stimuli as an adaptation to stress. The centre of the enthesis consisted of fibrocartilaginous tissue. The ligamentous part is highly vascularized and lacks shiny white fibres [18, 22]. The vascularization within the transition zone was lower than the root ligament, suggesting that the blood vessels supplying the ligament originate directly from the perimeniscal capillary ring and are not branches from the radial arteries from the meniscus red‐red zone, as in humans [15]. An anterior inter‐meniscal ligament (AIML) [30, 35] was never present. Previous reports either reported its presence [2], or corroborated its absence in 80% [47] or all examined specimen [48]. The ovine menisci are structurally similar to the human menisci [20], as their most peripheral region is highly vascularized and contains fibroblasts, while the inner region lacks blood vessels and is rich in chondrocyte‐like cells. The present study validates these findings [11], as vascularity, collagen structure and cellularity were not significantly different between human and sheep; making the ovine model a more preferable candidate for meniscal repair than the rabbit because of its structural similarities [11].

Even if the smaller proportions of sheep led to reduced dimensions [22, 30], the sheep is closest to humans when topographically comparing the tibial subchondral bone structure of pig, rabbit, rat and mouse [31, 45]. Also, its stifle joint differs from the human knee slightly in terms of leg alignment and range of motion, as the unguligrade sheep have a constitutional tibia valga and its resting position relative to the femur is in flexion, dissimilar to humans [39]. The load distribution of the ovine stifle joint is similar to humans during walking, with a higher load in the medial compartment of 60‐80% during the maximum load phase of [46] versus 60%–70% for humans [6, 28, 29, 46]. Similar to humans, the ovine MTP was of a significantly longer sagittal length than lateral [10, 41]. The aspect ratio of the ovine MTP is significantly higher than the LTP, while in humans the aspect ratio for the MTP is lower than for lateral [10]. Interestingly, the attachments and lengths of the MAR and LAR were significantly different. The MAR was significantly wider than the LAR, while in humans, the mean width of the MAR was reported to be 10.0 ± 1.5 mm, while the mean width of the LAR was 10.5 ± 1.4 mm [38]. Similar to humans (mean widths of MPR and LPR: 10.5 ± 1.0 mm and 11.4 ± 1.4 mm, resp [38]), the ovine LPR was wider than the MPR. The sheep medial meniscus body, posterior horn and MPR were all significantly thinner than lateral, while the lengths of the medial and lateral anterior root and widths of the anterior horns were not significantly different. In humans, the medial meniscus anterior horn (8.38 ± 1.64 mm) and meniscus body are thinner than lateral (anterior horn: 9.84 ± 1.78 mm). The human medial posterior horn is wider than the lateral posterior horn [33]. Remarkably, the widths of the ovine MAR and the LAR were approximately half of both anterior horns. This is in a striking contrast compared to humans [33, 38], where the widths of the MAR and the LAR are not different to those of their horns. These comparisons imply that strategies involving the medial compartment cannot simply be converted to the lateral compartment.

The ovine ACL has two different tibial entheses (lateral and medial) while humans have a singular one [30]. The human MAR is attached more anteriorly, closer to the TT along the anterior margin of the MTP, further away from the ACL footprint, traversing less laterally compared to sheep [22, 23, 30]. The ovine ACL TT may serve as key anatomical landmarks because of their proximity to the MARA. Similar human anatomical landmarks were established by LaPrade et al. [30]. The tibial eminence and tuberosity were strongly related to the MAR: the shorter the distance between the medial aspect of the tuberosity and the MARA, the longer the MAR will be; and the shorter the distance between the MARA and the centre of the tuberosity, the longer the distance between the MARA and the eminence will be. As the TT is a major anatomical structure, these data may be helpful for a safe surgical approach. Dimensions of the medial and lateral ‘bare areas’ adjacent to the MARA/LARA have not yet been described, to the best of our knowledge.

Limitations include that no biomechanical data were provided, data was solely collected once per specimen, and all specimen were of female animals, which should be considered when performing studies involving hormonal physiology. Strengths include the accurate quantitative information of meniscus‐relevant anatomical structures. The morphologic data of the present study may be helpful for strategies to test approaches for meniscus (and root) repair and replacement in sheep, to design ‘anatomic’ tissue‐engineered structures or prosthetic devices [13, 27, 34, 40].

From a clinical standpoint, it is well known that root and meniscus tears are associated with articular cartilage degeneration that will ultimately lead to knee OA [21, 49]. When aiming to establish an ovine model which could assess the natural history of meniscus or root tears and how they instigate knee OA [36], it is critical to first define the quantitative anatomy of the ovine menisci and their root attachments to determine if such a model faithfully reflects the human situation. The findings of the present study suggest the appropriateness of the sheep model for such translational approaches.

## CONCLUSION

The ovine MAR may be appropriate for repair approaches because of its morphological similarities to the human situation. The substantial differences between the medial and LTP have to be respected.

## AUTHOR CONTRIBUTIONS

Henning Madry conceived and designed the study. Material preparation, data collection and analysis were performed by Matthias Brockmeyer, Marta Carretero‐Hernández, Wei Liu, Henning Madry and Yin Zhang. Data were interpreted by Marta Carretero‐Hernández, Wei Liu, Henning Madry and Yin Zhang. All authors contributed to drafting and revising the manuscript and have approved the submitted version of the final manuscript.

## CONFLICT OF INTEREST STATEMENT

The authors declare no conflicts of interest.

## ETHICS STATEMENT

The authors have nothing to report.

## Supporting information

Supporting information.

## Data Availability

The authors have nothing to report.
