# Supplementary material for: A new preclinical sheep model of medial meniscus anterior root repair: Part 1—Quantitative morphology and relationships to adjacent structures
Source: Knee Surg Sports Traumatol Arthrosc. 2025 Mar 25;33(7):2470–86. doi: 10.1002/ksa.12656 (PMC12205422; doi:10.1002/ksa.12656)
Supplement: Supplementary file 1 — Supporting information. [file KSA-33-2470-s001.pdf]

A new preclinical sheep model of medial meniscus root repair. Part 2. Surgical strategy, technical considerations, pearls and pitfalls

Abstract

Purpose: To address a gap in translational research by developing a preclinical sheep model of medial meniscus anterior root (MAR) repair *in vivo* and to compare probabilities of potential pitfalls and difficulties with humans.

Methods: Preoperative planning and surgical procedure applied to patients were adapted to adult sheep. Eight healthy, skeletally mature, female Merino ewes between 2 and 4 years of age underwent a mini-open medial parapatellar approach to both stifle joints without luxating the patella. Next, the MAR was transected in 16 knees (8 sheep) resulting in a subtype 2A tear according to the LaPrade classification, followed by a transtibial pull-out repair through a 3.5 mm diameter bone tunnel with a reinforced Mason-Allen suture and non-absorbable suture material. Animals were followed until 21 days after surgery.

Results: The surgery time per knee ranged between 30 and 50 min (mean, 40.0 ± 7.8 min). The surgical technique was safe without intra- or postoperative complications. Solid repair is most likely if the following surgical principles are respected: (1) Selection of the MAR and the open technique allow for elegant tunnel positioning and less postoperative loading stress due to the normal extension deficit of sheep; (2) careful preparation of the MAR is mandatory; (3) considering the oval shape of the MAR attachment (MARA) results in anatomic tunnel placement; (4) robust suture placement and configuration avoids suture cut out. The probabilities of potential pitfalls and difficulties differ from the human situation.

Conclusion: A clinically adapted MAR repair model in adult sheep was developed following its complete transection close to the MARA, followed by an open transtibial pull-out repair. The surgical technique was safe without intra- or short-term

postoperative complications. This model may be suitable to study the biomechanics and pathophysiology of meniscal root injuries and their repair.

Level of evidence: 4

Keywords Preclinical large animal model, medial meniscus anterior horn root, root repair, surgical technique, sheep model.

Introduction

Meniscal root tears are defined as a complete avulsion of the meniscal root fibers connecting the meniscal tissue with the cortical bone of the tibial plateau or complete radial tear of all circumferential fibers of the meniscus tissue in direct proximity (10 mm) to the root attachment [2, 6, 19]. Meniscus root tears impede the protecting function of the menisci, resulting in increased joint loads equivalent to total meniscectomy [1, 2, 6, 19]. Large-animal models and clinical data suggest that root tears result in joint deterioration and early osteoarthritis (OA) [3, 14, 36]. The emerging picture over the past few decades suggests that in clinical practice, root tears are challenging to treat and meniscal root repair only partially restores the native biomechanics of the knee joints [2, 6, 17-19, 35]. However, recent systematic reviews suggest that meniscal root repair produces improvements in radiological and patient-reported outcomes superior to partial meniscectomy and nonoperative treatment [16, 25].

Translational large animal models of the knee help to understand associated pathologies and have led to the development of improved surgical treatment measures [3, 4, 26, 32]. Sheep are established model organisms [21], serving to improve surgical techniques [22, 29, 34, 37], avoiding possible pitfalls [32], and are especially valuable to understand the consequences of meniscal injuries including their roots to induce OA [27, 29], to test new treatment strategies for osteochondral defects [33] or other musculoskeletal disorders [12, 31].

So far, Dzidzishvili et al. established a rabbit model of medial meniscus posterior root (MMPR) release, showing that MMPR repair reduced but did not avoid the progression of OA, yet led to significantly less severe degenerative changes than partial meniscectomy and nonoperative treatment [13, 14]. Also in rabbits, a transtibial pull-

out repair of the medial meniscus anterior horn with an additional injection of autologous platelet-rich plasma gel into the bone tunnel was performed [10]. Because of their size, however, such pioneering small animal models of meniscus root repair enable only reduced insights into the effects of repair on the associated structures, for example on the specific topographical changes within the tibiofemoral compartment, complicating an accurate spatial assessment of OA development following root repair and/or application of novel regenerative therapies [29]. Recently, repairing MMPRTs was attempted in a goat model [11]. In order to achieve a highly precise and anatomic root repair in sheep, and to provide a convenient model that can be applied to investigate the effect of different repair and regenerative techniques on OA development which is nearly always induced in experimental settings by transecting the MAR [27-29], we focused on MAR repair in contrast to the posterior root which is much more prevalent in the clinical setting in humans and due to the smaller dimension of the stifle joint and the normal extension deficit of 30-40° compared to the human knee joint [32].

The objective of the present study was therefore to address a gap in translational research by developing a medial meniscus anterior root (MAR) repair model in sheep and to compare the surgical strategy, technical considerations and pitfalls with the clinical situation. Our hypothesis was that a safe sheep model of medial meniscus anterior root repair could be developed and that the probabilities of potential pitfalls and difficulties *in vivo* differ compared with humans due to anatomical differences.

Materials and Methods

Overview of the animal model

Skeletally mature sheep underwent a mini-arthrotomy of both stifle joints and subsequent transection of the medial meniscus anterior root followed by immediate repair using a reinforced Mason-Allen suture and non-absorbable suture material. The surgical procedures were performed by two experienced orthopaedic surgeons (MB and HM) with profound knowledge in open and arthroscopic knee surgery.

Animals for medial meniscus anterior root repair

Eight healthy, skeletally mature, female Merino sheep between 2 and 4 years of age received water ad libitum, were fed a standard diet (with a 12-h fast preoperatively) and were monitored at all times by specialized human and veterinary surgeons. After delivery to the animal facility, all animals were given a 14-day period to acclimatize. They were kept on straw in an outdoor enclosure in the fresh air, and completed an acclimatization program. Animal experiments were conducted in accordance with the local and national legislation on protection of animals and the NIH Guidelines for the Care and Use of Laboratory Animals. They were approved by the local governmental animal care committee (2.4.2.2 – 22-2023, Saarland, Germany).

Anesthesia

Xylazine (Elanco Tiergesundheit, Basel, Switzerland) 0.05 mg/kg body weight (b.w.) was administered i.v. via an ear vein cannula, followed by 2-5 mg/kg ketamine i.v. in a separate syringe (Serumwerk Bernburg, Bernburg, Germany). The animal was then carefully transported to the operating room and placed a supine position on the operating table. Intubation was performed using an endotracheal tube (tube size adapted to weight: 60-80 kg b.w.: 10-12 Ch; tube at least 40 cm long). Lidocaine

(Aspen, Munich, Germany) was applied to the tube to prevent laryngeal spasm. A rumen tube was inserted. Anesthesia was maintained with isoflurane (Piramal Critical Care, Voorschoten, Netherlands). The following ventilation parameters were applied: Frequency: 16-19 breaths, tidal volume: 10-15 ml/kg b.w., inspiratory pressure: 18 cm H_2O, expiratory pressure: 5 cm H_2O, tidal volume with 10-20 ml/kg b.w.. After attaching electrocardiogram (ECG) electrodes to the shaved chest, the intraoperative ECG including heart rate were recorded. Buprenorphine (CP-Pharma, Burgdorf, Switzerland) was administered intraoperatively as an analgesic. Intraoperatively, each animal received a single antibiotic prophylaxis with amoxicillin (30 mg/kg b.w.) (Bimeda Animal Health, Dublin, Ireland). In addition, NaCl 0.9% was slowly applied i.v. as fluid substitution throughout the entire operation. During the postoperative recovery phase, carprofen (Zoetis, Vechta, Germany) was always administered as non-steroidal anti-inflammatory drug (4 mg/kg b.w. i.m.). A bite block was used to prevent biting. The precise surgical technique of the root repair model is given in the Results section.

Immediate postoperative phase (4 weeks)

We monitored intraoperative complications such as injury to the patellar tendon, the medial meniscus anterior horn or its root, the anterior cruciate ligament (ACL) or anesthesiologic incidents. Additionally, we observed the immediate postoperative phase regarding postoperative return to fully weight-bearing and postoperative complications such as patella luxation. The surgery time was measured.

Comparison of the surgical strategy, technical considerations and pitfalls with the clinical situation

We compared the surgical strategy, technical considerations and probabilities of potential pitfalls and difficulties with the clinical situation in humans.

Statistical analysis

Data are given as the mean and standard deviation. All calculations were performed with Prism v.10.2.3 (GraphPad Software, San Diego, USA).

Results

Preparation of the surgical field

The forelimbs of the animals were secured, the entire lower extremity up to and including the groin was depilated by shaving the fur, and the feet and claws were completely wrapped with sterile adhesive drapes. The entire lower extremity (both hind legs) and the groin area were carefully disinfected three times. The groin area was covered with a sterile surgical drape and secured with additional adhesive drape to create an absolutely sterile surgical site.

Surgical approach to the ovine MAR

After a final disinfection, the medial femoral condyle, anterior edge of the medial tibial plateau, the tibial tuberosity and the patellar ligament were palpated, serving as anatomical landmarks (**Fig. 1 and 2**). All incisions were carried out as small as possible. An oblique skin incision of 3.5 – 4.0 cm length spanning medio-proximal from the lateral aspect of the medial femoral condyle over the anterior edge of the medial tibial plateau to approximately 2.0 cm latero-distal to the medial proximal edge of the tibial tuberosity was performed using a No. 20 scalpel knife with the stifle joint slightly (30°) flexed as a mini-open medial parapatellar approach without luxating the patella [17]. After the skin incision, the subcutaneous fat was incised with electrocautery and the medial retinaculum was visualized without exposing the patellar tendon. The medial retinaculum and joint capsule were then incised with electrocautery. To expose the medial meniscus anterior horn, MAR area (MARA) and ACL footprint, the prominent Hoffa fat pad was partially incised with electrocautery. Care has to be taken to avoid an accidental incision into the medial meniscus anterior root during the surgical exposure due to the prominent medial part of the Hoffa fat pad. Then, the lateral aspect of the medial femoral condyle, the medial meniscus anterior horn, the MAR and MARA

and both the anteromedial (AMB) and anterolateral bundle (ALB) of the ACL and their footprints were identified and prepared by careful dabbing. Incisions were kept open by inserting a self-retaining spreader and by means of small Hohmann and Langenbeck retractors. The medial meniscus anterior root was further exposed by slightly lifting it with a curved Overholt clamp from the medial tibial plateau after identifying its medial edge and carefully separating it at its entire length from any soft-tissue attachments. Small fibers that sometimes ran from the posterior part of the MAR to the medial retinaculum/Hoffa fat pad were also carefully transected. The exposed and released MAR was then transected 1-2 mm close to the MARA with a No. 15 scalpel knife, resulting in a complete medial MAR tear with a spontaneous separation of its edges of 2-4 mm.

Figure 1

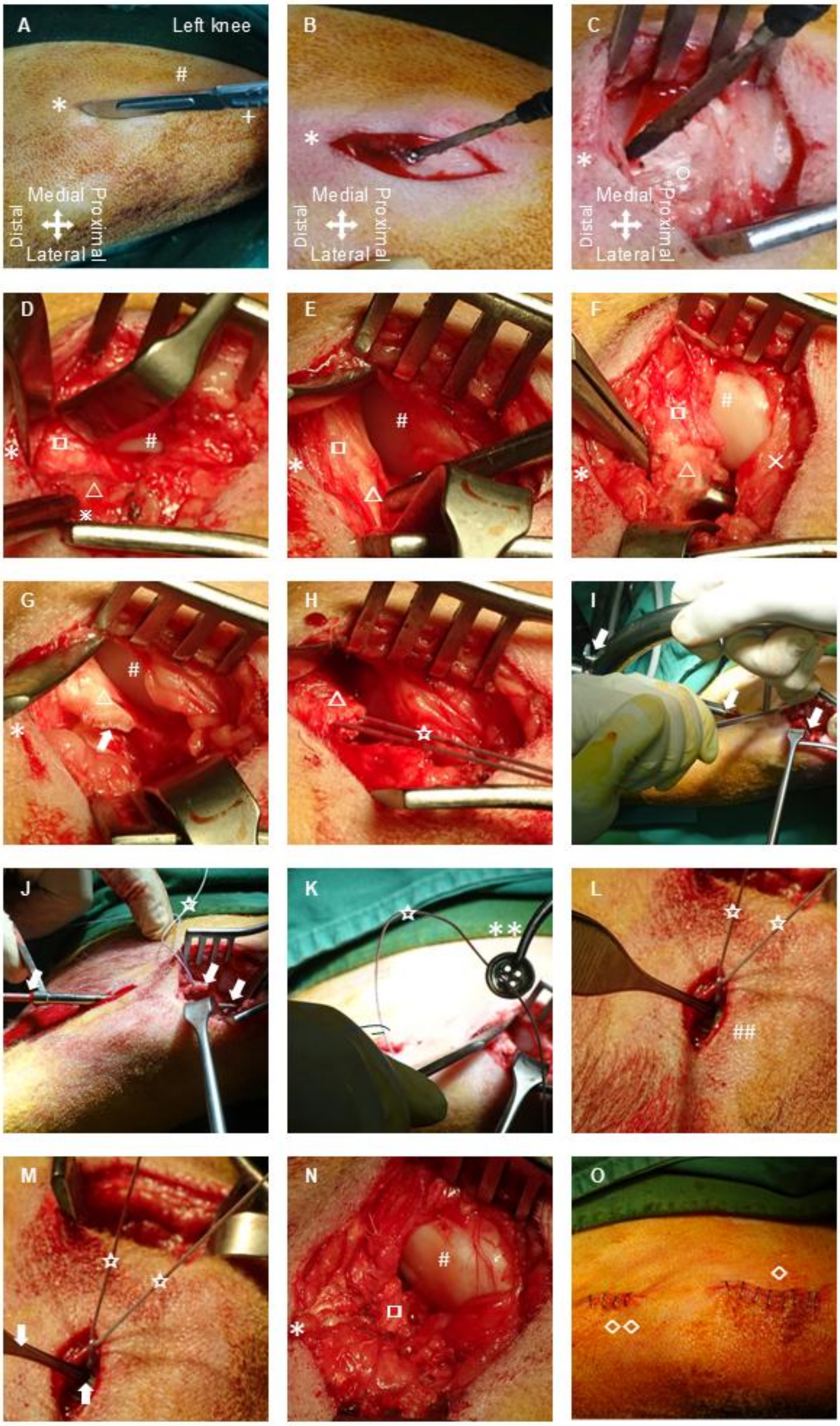
A
Left knee
Medial
Lateral
Distal
Proximal
B
C
D
E
F
G
H
I
J
K
L
M
N
O

Figure 2

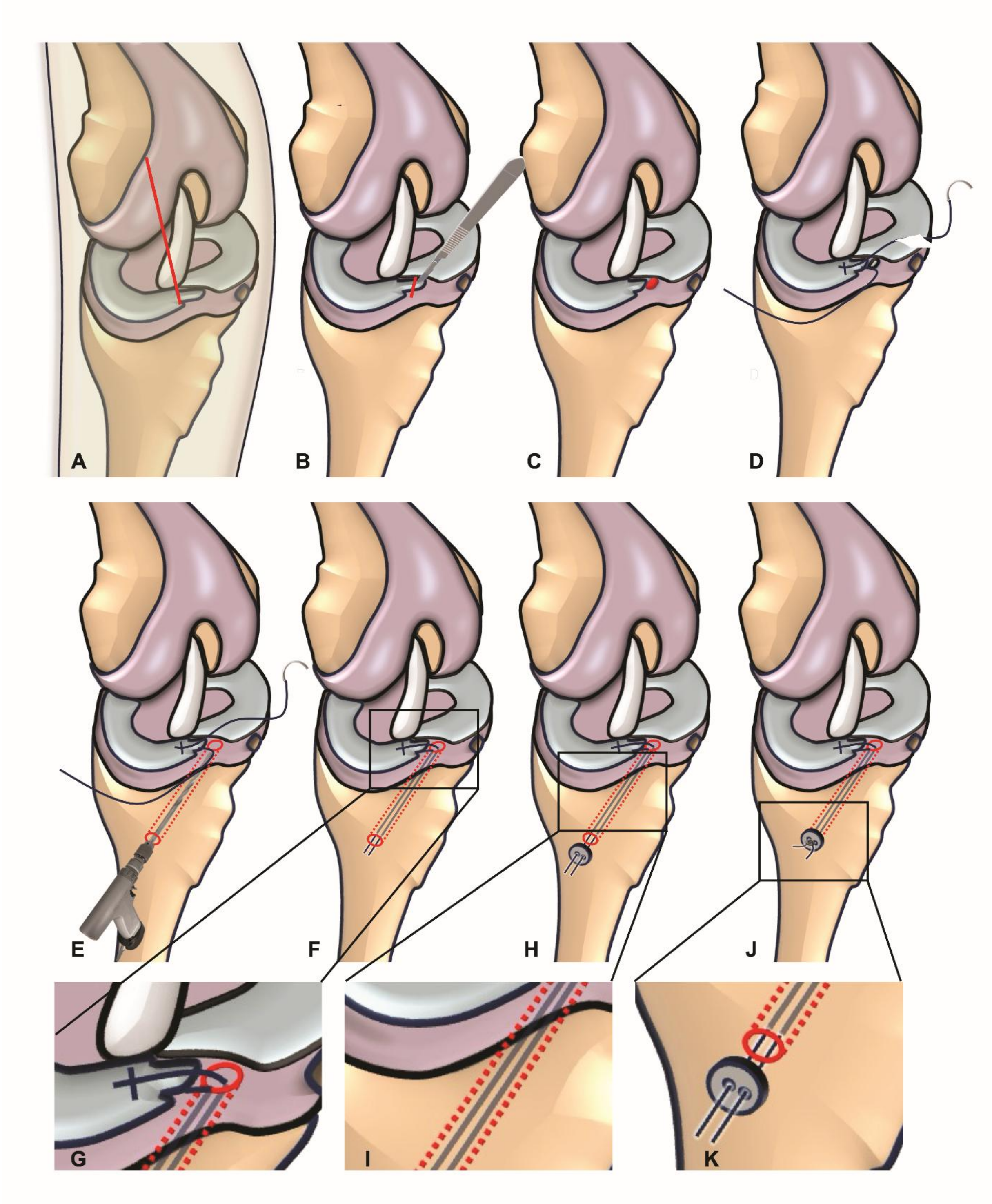

Surgical technique of MAR repair

The anterior horn of the medial meniscus was reinforced by a Mason-Allen suture (**Fig. 3**) using robust non-absorbable suture material (FiberWire®, Arthrex, Naples, FL, USA). The MARA was sufficiently decorticated using a curved curette to enhance the healing process of the meniscal root. The tip of a curved aimer guide was positioned at the desired site of the MARA area. Then, an additional small skin incision was established over the anteromedial tibia and the tip of a Kirschner wire serving as guide wire was placed through the aimer guide (set at 50° - 60°) from the anteromedial tibial cortex into the center of the MARA, about 35-40 mm distal from the medial tibial plateau. After verification of the correct guide wire placement, the wire was overdrilled by a 3.2 mm cannulated surgical drill (Synthes, Umkirch, Germany). The guide wire was then removed from the tibial bone tunnel and a rigid suture lasso (nitinol wire) was inserted through the cannulated drill that was left in place. The suture ends were captured and shuttled through the transtibial bone tunnel by the suture lasso. The cannulated drill was then removed. For the extraosseous suture fixation, a small suture disc was introduced. The knots were tied under direct visualization of the reinforced medial meniscus anterior horn to adjust the correct meniscus tension and sufficient reduction to its anatomic tibial position (MARA). The fixation was performed in a stifle position near extension between 40-50° of flexion. Finally, the adequate repair result was assessed by a palpating hook. The joint was then rinsed with sterile NaCl 0.9%, followed by layer-by-layer closure of the joint capsule and retinaculum using interrupted sutures (USP 2; Vicryl, Ethicon, Johnson & Johnson, Norderstedt, Germany) and subcutaneous tissue using an inverted interrupted suture (USP 2.0; Vicryl, Ethicon). The skin incision was closed with surgical staples (Covidien Appose, Medtronic, Meerbusch, Germany), then additionally disinfected and covered with a spray dressing (Aluminium-Spray, Pharmamedico, Twistringen, Germany). The total

operating time of each surgery was determined from the first incision to the last suture stitch. An overview of surgical pearls is presented in **Table 1**.

Figure 3

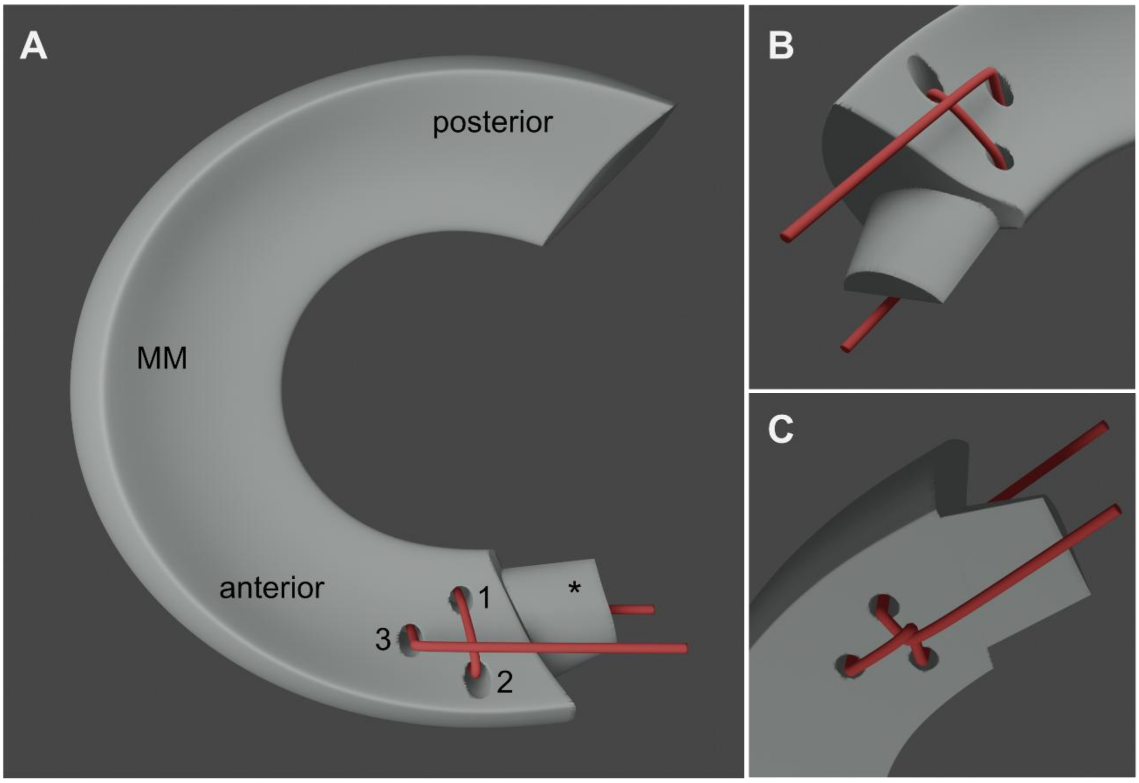

Immediate postoperative phase (4 weeks)

No intraoperative complications occurred, the patellar tendon, the medial meniscus anterior horn or its root or the anterior cruciate ligament were never injured and the anesthesia was always uneventful. The surgery time per knee (n = 16 knees; 8 sheep) ranged between 30 and 50 minutes (mean, 40.0 ± 7.8 minutes). Two hours after the procedure, all sheep were fully weight-bearing. At 21 days postoperatively, one animal developed an anteromedial superficial skin infection that remained extraarticular and was successfully conservatively treated with local antiseptic measures. No patella luxation or other postoperative complications occurred. All incisions healed after 2–3 weeks.

Comparison of the surgical strategy, technical considerations and pitfalls with the clinical situation

Anatomical parameters that are relevant for MAR repairs and that differ between sheep and humans were identified. The resulting surgical consequences are presented in Table 2 and probabilities of potential pitfalls and difficulties among sheep and humans in Table 3.

Discussion

This study developed a MAR repair model in adult sheep. The MAR was transected close to the MARA resulting in a LaPrade subtype 2A tear, followed by transtibial pull-out repair with a reinforced Mason-Allen suture and non-absorbable suture material, followed by an open transtibial pull-out repair. Solid repair is most likely if the following surgical principles are respected: (1) Selection of the MAR and open technique allow for elegant tunnel positioning; (2) careful preparation of MAR is mandatory; (3) considering the oval shape of the MAR attachment results in anatomic tunnel placement; (4) robust suture placement and configuration may prevent suture cut out. The surgical technique was safe without intra- or immediate postoperative complications related to MAR release and repair. It was compared within the context of potential pitfalls and difficulties with humans.

The presented model of MAR transection (close to the MARA) represents a complete MAR tear classified as subtype 2A according to LaPrade et al. [24]. Steineman et al. performed an arthroscopic technique to transect the medial and lateral meniscus anterior horn attachment in adult Flemish Giant rabbits and left them untreated without root repair, confirming early OA development [36]. Dzidzishvili et al. used New Zealand white rabbits, where after medial meniscus posterior root release, early and severe OA changes emerged at 16 weeks post-surgery [14]. In goats, MMPRTs lead to severe OA changes at 12 and 24 at 16 weeks post-surgery [11]. A posteromedial approach between the medial head of the gastrocnemius and the medial collateral ligament (MCL) was selected for arthrotomy and to transect the posterior root of the medial meniscus [14]. Bansal et al. [3, 4] developed an arthroscopic meniscal injury model in Yucatan minipigs. In three different experimental groups, they performed a sham surgery (arthroscopic meniscal visualization), introduced an acute arthroscopic vertical

defect in the anterior horn or an acute arthroscopic transection of the anterior horn attachment of the MAR to simulate a MAR tear. Interestingly, the authors found a spontaneous reattachment (maturation of a fibrovascular scar) of the anterior horn after transection. Their work underlined the risk of early onset of OA after clinically relevant meniscal tears [3]. Sheep models of MAR release allowed to perform detailed topographic modeling of human early OA [29] and to study the effect of high tibial osteotomy on knee OA development [30].

So far, only a few studies attempted meniscus root repair, and most of them were performed in small animal models. Comparable to the present study, Cui et al. [10] succeeded in a transtibial pull-out repair of the medial meniscus anterior horn to treat meniscal root tears in rabbits. The surgical approach to the stifle joint was performed in a comparable, open manner to our technique and the MAR was also transected close to the MARA. The authors used a similar transtibial pull-out repair technique except for the tibial fixation using a self-made steel wire. Additionally, and in contrast to our methods, they injected autologous platelet-rich plasma gel to the bone tunnel. The authors described early healing of the meniscus and bone postoperatively, and proposed that this treatment may reduce the risk of secondary cartilage defects. In a study based on their previously described rabbit model, Dzidzishvili et al. [13] found that meniscus root repair did not fully stop the progression of knee OA but had significantly less severe degenerative changes than partial meniscectomy and nonoperative treatment. The root repair was conducted in a comparable fashion with those of our study, using also an open surgical approach and the transtibial pull-out technique, while performing a MMPRT repair and using a different tibial suture fixation method (suture tied out over the cortical bone on the tibia). Recently, MMPRTs were repaired in a goat model using transosseous sutures, showing incomplete healing and

persistent medial meniscal extrusion after 24 weeks, based on discontinuities in the root-meniscus transition, underscoring the importance of this junction in the context of MMPRT repair [11]. Here, the repair of the MAR was conducted because the approach to and the precise visualization of the anterior root is more reproducible and careful compared with the exposition of the posterior root. This might prevent iatrogenic damage to the adjacent intraarticular structures especially the osteochondral unit. The area of MARA is described as the largest meniscal insertion site in humans [20], therefore a similar size ratio was assumed here. All sheep had an insertion type-I based on the Berlet et al. classification, flat in the intercondylar region of the tibial plateau [5]. Therefore, the use of the medial meniscus anterior horn in the present study might enhance a more precise anatomic tibial tunnel placement in the MARA during the root repair as the posterior horn.

Compared to the human knee joint, the approach to the MAR was more challenging due to the smaller dimension of the sheep stifle joint and therefore, an open access via a medial parapatellar arthrotomy was selected. Of note, some anatomical parameters that are relevant for medial meniscus anterior root repairs differ between sheep and humans, resulting in surgical consequences (**Table 2**) and potential pitfalls (**Table 3**). After the arthrotomy, the visualization of the root attachment appeared easier in sheep since their root attachment is localized more posterior to the tibial tuberosity and nearer to the ACL footprints compared to the human knee, increasing the risk of inadvertent damage to the ACL during exposure and tunnel drilling. The fiber structure of the medial meniscus anterior horn and its root was less robust and more fibrous compared to humans, making the reinforcement more difficult. Therefore, it was decided to use the Mason-Allen stitching technique and to place the suture through the anterior horn to ensure a maximum primary suture stability. Additionally, the mobility of

the sheep medial meniscus anterior horn was more restricted than the human one, so that the anatomic reduction was more demanding. In summary, the most relevant anatomical differences between sheep stifle and human knee joints are a smaller dimension and a normal extension deficit of the stifle joint and a closer relation to the ACL compared to human knee joints. This makes a direct transfer to the human clinical settings difficult as different joint kinematics and loads might be present. In addition, we found further differences with regard to the fiber structure of the medial meniscus anterior horn and its properties. The present sheep model focused on the medial meniscus anterior horn, a similar location than many experimental studies of topographical OA development [27, 29]. This might also influence the generalizability to human clinical conditions involving medial meniscus posterior roots.

The current investigation is not without limitations. The major one is the fact that the presented model uses the anterior root while clinically the posterior root is much more common which raises translational challenges. However, we have recently confirmed the high structural similarity between the ovine anterior and posterior regions [29]. Moreover, surgical transection of the posterior root may require a temporary release of the MCL, affecting stability, thus also the root repair result and the course of OA. Also, it is possible that the load on the posterior roots is higher as the sheep have a normal extension deficit of about 30-40°, thus may have been more detrimental as the animals were not allowed for ethical reasons to unload their legs postoperatively, leading to strong biomechanical forces and consequently a possible failure of reconstruction might be possible. This is in contrast to current postoperative treatment recommendations after meniscal root repair in patients, potentially affecting translational validity [9]. The model´s direct applicability may be restricted by differences in biomechanical properties and joint geometry between sheep and

humans. Second, because of reported differences in the ovine mini-open technique and arthroscopic surgical repair techniques (not performed here) and instrumentation used in a clinical situation, it is difficult to draw strict conclusions on its direct comparability. The use of a single tunnel design for transtibial pull-out repair may less restore the complex loading patterns of the meniscus compared with a double tunnel technique. While the type of tear induced here represents a traumatic tear, a considerable number of patients suffers from root tears within the continuum of meniscus extrusion and OA changes. The study also does not address how the repair withstands cyclic loading or biomechanical stresses typical of knee joint motion, necessitating assessments of long-term mechanical integrity. Finally, the short-term outcomes (21 days postoperative) address surgical feasibility and safety effectively, but mid- to long-term functional results [11] healing and OA development are mandatory. Strengths include the first description of a sheep model for immediate MAR repair of a relevant (LaPrade subtype 2A) root tear and suture technique, highlighting in detail the surgical strategy, technical considerations, pearls and pitfalls in clinically adapted fashion.

In the future, this sheep model of MAR repair can be applied to study the effect of different suture techniques, varying locations of meniscus or root damage, testing local or injectable cell-free or cell-based regenerative therapies, different natural and synthetic scaffolds for meniscal repair or replacement or varying biomechanical loading, all with a view on the development and prevention of knee OA, reflecting the clinical situation.

Conclusion

A MAR repair model in adult sheep was developed following complete transection of the MAR close to the MARA, representing a subtype 2A according to the LaPrade classification followed by an open transtibial pull-out repair. The surgical technique was safe without intra- or short-term postoperative complications.

Abbreviations

Anterior cruciate ligament	ACL
Anterior cruciate ligament, anterolateral bundle	ALB
Anterior cruciate ligament, anteromedial bundle	AMB
Electrocardiogram	ECG
Lateral meniscus anterior root	LAR
Medial collateral ligament	MCL
Medial meniscus	MM
Medial meniscus anterior root	MAR
Medial meniscus anterior root attachment	MARA
Medial meniscus posterior root	MMPR
Osteoarthritis	OA

Figure Legends

Figure 1

Surgical technique of medial meniscus anterior root repair in sheep (left knee). (**A**) Skin incision for the mini-open medial parapatellar approach without luxating the patella (+: patella, #: medial femoral condyle, *: medial tibial plateau). (**B**) Subcutaneous incision electrocautery (*: medial tibial plateau). (**C**) Incision of the medial retinaculum and capsule with electrocautery (*: medial tibial plateau, ○: medial retinaculum). (**D**) Without luxating the patella, the medial meniscus anterior horn, MAR and ACL footprint are exposed (#: medial femoral condyle, *: medial tibial plateau, □: medial meniscus anterior horn, □: MAR, ※: ACL footprint). (**E**) Detailed visualization of the medial meniscus anterior horn and MAR (#: medial femoral condyle, *: medial tibial plateau, □: medial meniscus anterior horn, □: MAR). (**F**) The MAR is lifted with a curved Overholt clamp from the medial tibial plateau and transected (#: medial femoral condyle, *: medial tibial plateau, □: medial meniscus anterior horn, □: MAR, ╳: Hoffa fat pad). (**G**) After transection of the MAR close to the MARA, the edges spontaneously separate (arrow) (#: medial femoral condyle, *: medial tibial plateau, □: MAR). (**H**) Mason-Allen suture using robust non-absorbable material (□: MAR, ☆: suture). (**I**) Placement of the aimer guide (arrows). (**J**) Overdrilling of the guide wire with a 3.2 mm cannulated surgical burr. (arrows: burr, ☆: suture material) (**K**) Suture disc insertion after shuttling of the suture ends through the transtibial bone tunnel (**: suture disc, ☆: suture material). (**L**) Placement of the suture disc directly on the anteromedial cortex of the tibia (##: tibia cortex, ☆: suture). (**M**) Assisted suture tying using a surgical forceps (arrows: forceps, ☆: suture). (**N**) Control of the final repair result (#: medial femoral condyle, *: medial tibial plateau, □: reduced medial meniscus anterior horn). (**O**) Closure of the mini-open arthrotomy and additional incision for the suture disc with surgical staples (◇**:** Closure of the mini-open arthrotomy**,** ◇◇**:** Closure of the incision

for the suture disc). ACL: anterior cruciate ligament; MAR: medial meniscus anterior root; MARA: medial meniscus anterior root attachment.

Figure 2

Schematic drawing of the surgical key steps for medial meniscus anterior root repair in sheep. (**A**) Anteromedial parapatellar arthrotomy (red line). (**B**) Transection of the medial meniscus anterior horn root (simulated root tear, red line). (**C**) Preparation of the medial meniscus anterior horn root area (bony debridement using curette, red area). (**D**) Placement of a Mason-Allen stitch at the medial meniscus anterior horn using non-absorbable suture material. (**E**) Creating a 3.5 mm bone tunnel from the anteromedial tibial cortex ending in the center of the medial meniscus anterior horn root using aiming device and guide wire. (**F**) Transtibial pullout of suture ends using a rigid suture lasso. (**G**) Adjusting the correct meniscus tension under direct visualization of the reinforced medial meniscus anterior horn (enlarged picture detail). (**H**) Placement of a suture disc on the tibial cortex at the end of the suture. (**I**) Transtibial suture pullout through a 3.5 mm bone tunnel (enlarged picture detail). (**J**) Extraosseous suture fixation by a suture disc. (**K**) Final result of tibial fixation after knot tying (enlarged picture detail).

Figure 3

Schematic drawing of the Mason-Allen suture (1: first stitch, 2: second stitch, 3: third stitch, MM: medial meniscus, *: medial meniscus anterior root). (**A**) View of the surface of the medial meniscus anterior horn. (**B**) View of the undersurface of the medial meniscus anterior horn. The Mason-Allen stitch: The first stitch (1) begins from the undersurface of the medial meniscus anterior horn through the meniscus tissue to its surface leaving sufficient tissue to the meniscus edges. The second stitch (2) starts from the surface of the medial meniscus anterior horn through the meniscus tissue to its undersurface leaving sufficient tissue to the meniscus edges and a sufficient tissue bridge between stitch 1 and 2. Then, the suture ends were crossed at the undersurface of the medial meniscus anterior horn and the third stitch (3) is placed more medially behind the suture crossing. The stitch passes from the undersurface of the medial meniscus anterior horn to its surface. MM, Medial meniscus.

Table 1

Surgical pearls of the ovine model and associated troubleshooting strategies.

- Avoiding a large incision of the medial retinaculum prevents a fatal postoperative patella luxation. Troubleshooting strategy: In case of extended incision of the medial retinaculum ensure a most accurate retinaculum suture with medial reefing.
- A sufficient surgical exposure helps to identify the root and adjacent structures, preventing possible accidental (partial) transection of adjacent structures, especially be aware of the close relation to ACL, LAR and medial femoral condyle. Troubleshooting strategy: In case of insufficient surgical exposure focus on a thorough resection of the prominent medial part of the Hoffa fat pad.
- The release of the MAR is facilitated by lifting it with an Overholt clamp. Troubleshooting strategy: In case of inadequate release and unfeasible lifting of the MAR carefully transect further small fibers that sometimes ran from the posterior part of the MAR to the medial retinaculum/Hoffa fat pad to better identify the medial edge of the MAR.
- The MAR is completely transected, including also small fibrous structures possibly extending from the MAR to the ACL or other structures. Troubleshooting strategy: In case of incomplete transection of the MAR search for small additional fibrous structures in relation to the MAR and transect them properly.
- The anatomical footprint of the MARA is accurately prepared, allowing for a precise placement of the tibial tunnel. Troubleshooting strategy: In case of inaccurately prepared footprint of the MARA do not compromise and start the tunnel drilling without further footprint preparation.

- Pay attention to a most accurate and robust Mason-Allen suture passing reliably through the meniscal tissue of the anterior horn (not through the MAR) to avoid suture tear out. Troubleshooting strategy: In case of inadvertent suture placement through the MAR or repeated suture cut-out place an additional suture more medially through the meniscal tissue of the anterior horn and check the suture strength with manual traction.
- Take time to adjust the correct root repair tension under direct visual and tactile control avoiding over- or undertensioning. Troubleshooting strategy: In case of repair undertensioning repeat the fixation procedure with more tension and check for a sufficient root reduction. In case of repair overtensioning check for a potential tunnel malplacement and place a new drill hole, if required.
- Avoiding soft tissue bridges between suture disc and anteromedial tibial cortex reduces the risk of secondary loss of reduction and tension. Troubleshooting strategy: Be aware of soft tissue bridges and prepare the anteromedial tibial cortex thoroughly by removing the soft tissue before placing the suture disc.
- After pulling out the sutures from the tunnel, assisted suture tying helps to securely fix the suture. Troubleshooting strategy: In case of loose suture tying and resulting root undertensioning repeat the fixation procedure with more tension, use the assisted suture tying (avoid using sharp clamps) and check for a sufficient root reduction.
- Final assessment of the adequate repair result by a palpating hook is mandatory.

Table 2

Comparison of relevant surgical anatomical parameters that are relevant for medial meniscus anterior root repair in sheep and humans.

Congenital anatomical differences	Sheep stifle joint	Human knee	Surgical consequence for the root repair model
**Knee range of motion: Extension/Flexion [°]**	0-40-130	5(-10)-0-120(-150)	Use of medial meniscus anterior root results in less postoperative loading stress due to the normal extension deficit of sheep
**Anatomical landmarks: closest relation**	ACL (anteromedial bundle)	ACL [23]	Most careful preparation of MAR due to close relation to ACL mandatory
**Biomechanical properties of the medial meniscus anterior root**	Ultimate failure strength [N]: 572.6 Stiffness [N/mm]: 143.5 [21]	Ultimate failure strength [N]: 655.5 Stiffness [N/mm]: 124.9 [15]	Relevant biomechanical aspects for root repair
**Shape of MARA**	Oval shaped; located anteromedial to the LAR attachment, and marginally anterior and lateral to the tibial ACL footprint	Four different tibial insertion locations according to Berlet et al.[5]: type I 59%; type II 24%; type III 15%; and type IV 3%	Information on the shape of the MARA is important for a reliable and anatomic tibial tunnel placement
**Approach / Bone tunnel placement**	Mini-open approach without patella luxation. Aimer guide, tunnel diameter: 3.2 mm, one tunnel	Arthroscopic, aimer guide, tunnel diameter: 4.5 mm, one or two tunnels [6, 8]	Open technique and anterior root allow an easier, more precise and anatomic tunnel placement
**Suture placement / technique**	Meniscus anterior horn / Mason-Allen stitch	Meniscus posterior horn / modified Mason-Allen stitch	Robust suture placement and configuration to avoid suture cut out
**Peak femorotibial force during walking**	2.27 x body weight [21]	2.1 to 2.7 x body Weight [7]	Relevant for postoperative rehabilitation, outcome and failure

ACL: anterior cruciate ligament, LAR: lateral meniscus anterior root, MAR: medial meniscus anterior root, MARA: medial meniscus anterior root attachment, N: Newton.

Table 3

Comparison of probabilities of potential pitfalls and difficulties among sheep and humans.

Structure involved	Sheep	Human
**Accidental (partial) transection of the medial meniscus anterior root during its surgical exposure due to the prominent Hoffa fat pad**	+/-	-
**Accidental (partial) transection of the ACL during the surgical exposure due to the prominent Hoffa fat pad**	+/-	-
**Accidental incision into the articular cartilage of the femoral condyle during its surgical exposure due to the prominent Hoffa fat pad**	+/-	-
**Irritation of the infrapatellar branch of the Nervus saphenus**	-	+/-
**Approach and identification of the anatomic location of the root**	+/-<br>Hoffa fat pad obscures the anatomical landmarks	+<br>Portal placement<br>Root visualisation in tight joints
**Release of other structures**	+<br>Fibers running from the MAR to the ACL and other anterior structures	+<br>Medial collateral ligament release
**Anatomic Tunnel Placement**	+/-	+
**Suture Configuration**	+/-	+
**Tension and Fixation**	+	+
**Rehabilitation**	+<br>Non-weight bearing difficult/impossible due to ethical considerations of animal welfare	-<br>Non-weight-bearing advised for the first 6 weeks

Human pitfalls refer to the arthroscopic approach. “+”: high probability, “+/-“: moderate probability, ”–“: low probability; ACL: anterior cruciate ligament, MAR: medial meniscus anterior root.

References

1. Allaire R, Muriuki M, Gilbertson L, Harner CD (2008) Biomechanical consequences of a tear of the posterior root of the medial meniscus. Similar to total meniscectomy. J Bone Joint Surg Am 90:1922-1931
2. Banovetz MT, Roethke LC, Rodriguez AN, LaPrade RF (2022) Meniscal Root Tears: A Decade of Research on their Relevant Anatomy, Biomechanics, Diagnosis, and Treatment. Arch Bone Jt Surg 10:366-380
3. Bansal S, Meadows KD, Miller LM, Saleh KS, Patel JM, Stoeckl BD, et al. (2021) Six-Month Outcomes of Clinically Relevant Meniscal Injury in a Large-Animal Model. Orthop J Sports Med 9:23259671211035444
4. Bansal S, Miller LM, Patel JM, Meadows KD, Eby MR, Saleh KS, et al. (2020) Transection of the medial meniscus anterior horn results in cartilage degeneration and meniscus remodeling in a large animal model. J Orthop Res 38:2696-2708
5. Berlet GC, Fowler PJ (1998) The anterior horn of the medical meniscus. An anatomic study of its insertion. Am J Sports Med 26:540-543
6. Bhatia S, LaPrade CM, Ellman MB, LaPrade RF (2014) Meniscal root tears: significance, diagnosis, and treatment. Am J Sports Med 42:3016-3030
7. Brzezinski A, Ghodbane SA, Patel JM, Perry BA, Gatt CJ, Dunn MG (2017) The Ovine Model for Meniscus Tissue Engineering: Considerations of Anatomy, Function, Implantation, and Evaluation. Tissue Eng Part C Methods 23:829–841
8. Chahla J, Moulton SG, LaPrade CM, Dean CS, LaPrade RF (2016) Posterior Meniscal Root Repair: The Transtibial Double Tunnel Pullout Technique. Arthrosc Tech 5:e291-296
9. Cong T, Reddy RP, Hall AJ, Ernazarov A, Gladstone J (2024) Current Practices for Rehabilitation After Meniscus Repair: A Survey of Members of the American

Orthopaedic Society for Sports Medicine. Orthop J Sports Med 12:23259671231226134

10. Cui P, Sun B-H, Dai Y-F, Cui T-Y, Sun J-L, Shen K, et al. (2023) Healing of the Torn Anterior Horn of Rabbit Medial Meniscus to Bone after Transtibial Pull-Out Repair and Autologous Platelet-Rich Plasma Gel Injection. Orthop Surg 15:617–627

11. Deng XH, Wang JS, Chen Z, Zeng WK, Peng HM, Yan WagiT, et al. (2024) Incomplete Histologic Healing and Diminished Biomechanical Strength of Meniscus-Bone Interface After Medial Meniscus Posterior Root Transosseous Repair in a Goat Model. Arthroscopy; S0749-8063(24)00552-8

12. Dominguez Perez JM, Fernandez-Sarmiento JA, Aguilar Garcia D, Granados Machuca MDM, Morgaz Rodriguez J, Navarrete Calvo R, et al. (2019) Cartilage regeneration using a novel autologous growth factors-based matrix for full-thickness defects in sheep. Knee Surg Sports Traumatol Arthrosc 27:950-961

13. Dzidzishvili L, Calvo E, López-Torres II (2023) Medial Meniscus Posterior Root Repair Reduces but Does Not Avoid Histologic Progression of Osteoarthritis: Randomized In Vivo Experimental Study in a Rabbit Model. Am J Sports Med 51:2964–2974

14. Dzidzishvili L, Lopez T, II, Guerrero CC, Calvo E (2022) Developing an experimental model of early knee osteoarthritis after medial meniscus posterior root release: an in vivo study. J Exp Orthop 9:66

15. Ellman MB, LaPrade CM, Smith SD, Rasmussen MT, Engebretsen L, Wijdicks CA, et al. (2014) Structural Properties of the Meniscal Roots. Am J Sports Med 42:1881-1887

16. Eseonu KC, Neale J, Lyons A, Kluzek S (2022) Are Outcomes of Acute Meniscus Root Tear Repair Better Than Debridement or Nonoperative Management? A Systematic Review. Am J Sports Med 50:3130-3139

17. Floyd ER, Rodriguez AN, Falaas KL, Carlson GB, Chahla J, Geeslin AG, et al. (2021) The Natural History of Medial Meniscal Root Tears: A Biomechanical and Clinical Case Perspective. Front Bioeng Biotechnol 9:744065

18. Garcia JR, Ayala SG, Allende F, Mameri E, Haynes M, Familiari F, et al. (2024) Diagnosis and Treatment Strategies of Meniscus Root Tears: A Scoping Review. Orthop J Sports Med 12:23259671241283962

19. Hantouly AT, Aminake G, Khan AS, Ayyan M, Olory B, Zikria B, et al. (2024) Meniscus root tears: state of the art. Int Orthop 48:955-964

20. Johnson DL, Swenson TM, Livesay GA, Aizawa H, Fu FH, Harner CD (1995) Insertion-site anatomy of the human menisci: gross, arthroscopic, and topographical anatomy as a basis for meniscal transplantation. Arthroscopy 11:386-394

21. Kohn D (1993) Autograft meniscus replacement: experimental and clinical results. Knee Surg Sports Traumatol Arthrosc 1:123-125

22. Kohn D, Wirth CJ, Reiss G, Plitz W, Maschek H, Erhardt W, et al. (1992) Medial meniscus replacement by a tendon autograft. Experiments in sheep. J Bone Joint Surg Br 74:910-917

23. LaPrade CM, Ellman MB, Rasmussen MT, James EW, Wijdicks CA, Engebretsen L, et al. (2014) Anatomy of the anterior root attachments of the medial and lateral menisci: a quantitative analysis. Am J Sports Med 42:2386-2392

24. LaPrade CM, James EW, Cram TR, Feagin JA, Engebretsen L, LaPrade RF (2015) Meniscal root tears: a classification system based on tear morphology. Am J Sports Med 43:363-369

25. Lee DR, Lu Y, Reinholz AK, Till SE, Lamba A, Saris DBF, et al. (2024) Root Repair Has Superior Radiological and Clinical Outcomes Than Partial Meniscectomy and Nonoperative Treatment in the Management of Meniscus Root Tears: A Systematic Review. Arthroscopy;10.1016/j.arthro.2024.02.017

26. Madry H, Ochi M, Cucchiarini M, Pape D, Seil R (2015) Large animal models in experimental knee sports surgery: Focus on clinical translation. J Exp Orthop 2:9

27. Olah T, Cucchiarini M, Madry H (2023) Subchondral bone remodeling patterns in larger animal models of meniscal injuries inducing knee osteoarthritis - a systematic review. Knee Surg Sports Traumatol Arthrosc 31:5346-5364

28. Olah T, Cucchiarini M, Madry H (2024) Temporal progression of subchondral bone alterations in OA models involving induction of compromised meniscus integrity in mice and rats: A scoping review. Osteoarthritis Cartilage 32:1220-1234

29. Olah T, Reinhard J, Gao L, Haberkamp S, Goebel LKH, Cucchiarini M, et al. (2019) Topographic modeling of early human osteoarthritis in sheep. Sci Transl Med 11:eaax6775

30. Olah T, Reinhard J, Laschke MW, Goebel LKH, Walter F, Schmitt G, et al. (2022) Axial alignment is a critical regulator of knee osteoarthritis. Sci Transl Med 14:eabn0179

31. Olthof MGL, Hasler A, Valdivieso P, Flück M, Gerber C, Gehrke R, et al. (2024) Poly(ADP-Ribose) Polymerases-Inhibitor Talazoparib Inhibits Muscle Atrophy

and Fatty Infiltration in a Tendon Release Infraspinatus Sheep Model: A Pilot Study. Metabolites 14:187

32. Pape D, Madry H (2013) The preclinical sheep model of high tibial osteotomy relating basic science to the clinics: Standards, techniques and pitfalls. Knee Surg Sports Traumatol Arthrosc 21:228–236

33. Peifer C, Oláh T, Venkatesan JK, Goebel L, Orth P, Schmitt G, et al. (2024) Locally Directed Recombinant Adeno- Associated Virus-Mediated IGF-1 Gene Therapy Enhances Osteochondral Repair and Counteracts Early Osteoarthritis In Vivo. Am J Sports Med 52:1336–1349

34. Stachel N, Orth P, Zurakowski D, Menger MD, Laschke MW, Cucchiarini M, et al. (2022) Subchondral Drilling Independent of Drill Hole Number Improves Articular Cartilage Repair and Reduces Subchondral Bone Alterations Compared With Debridement in Adult Sheep. Am J Sports Med 50:2669-2679

35. Steineman BD, LaPrade RF, Haut Donahue TL (2022) Loosening of Posteromedial Meniscal Root Repairs Affects Knee Mechanics: A Finite Element Study. J Biomech Eng 144:051003

36. Steineman BD, LaPrade RF, Santangelo KS, Warner BT, Goodrich LR, Haut Donahue TL (2017) Early Osteoarthritis After Untreated Anterior Meniscal Root Tears: An In Vivo Animal Study. Orthop J Sports Med 5:2325967117702452

37. Strauss E, Caborn DNM, Nyland J, Horng S, Chagnon M, Wilke D (2019) Tissue healing following segmental meniscal allograft transplantation: a pilot study. Knee Surg Sports Traumatol Arthrosc 27:1931-1938
